# Microbial osteoporosis: The interplay between the gut microbiota and bones via host metabolism and immunity

**DOI:** 10.1002/mbo3.810

**Published:** 2019-04-18

**Authors:** Lishan Li, Shitao Rao, Yanzhen Cheng, Xiaoyun Zhuo, Caihong Deng, Ningning Xu, Hua Zhang, Li Yang

**Affiliations:** ^1^ Department of endocrinology and metabolism Zhujiang Hospital Southern Medical University Guangzhou China; ^2^ School of Biomedical Sciences CUHK Shatin, N.T Hong Kong SAR China

**Keywords:** bone formation, bone resorption, intestinal microbiota, osteoporosis

## Abstract

The complex relationship between intestinal microbiota and host is a novel field in recent years. A large number of studies are being conducted on the relationship between intestinal microbiota and bone metabolism. Bone metabolism consisted of bone absorption and formation exists in the whole process of human growth and development. The nutrient components, inflammatory factors, and hormone environment play important roles in bone metabolism. Recently, intestinal microbiota has been found to influence bone metabolism *via* influencing the host metabolism, immune function, and hormone secretion. Here, we searched relevant literature on Pubmed and reviewed the effect of intestinal microbiota on bone metabolism through the three aspects, which may provide new ideas and targets for the clinical treatment of osteoporosis.

## 1 INTRODUCTION

**Figure 1:**
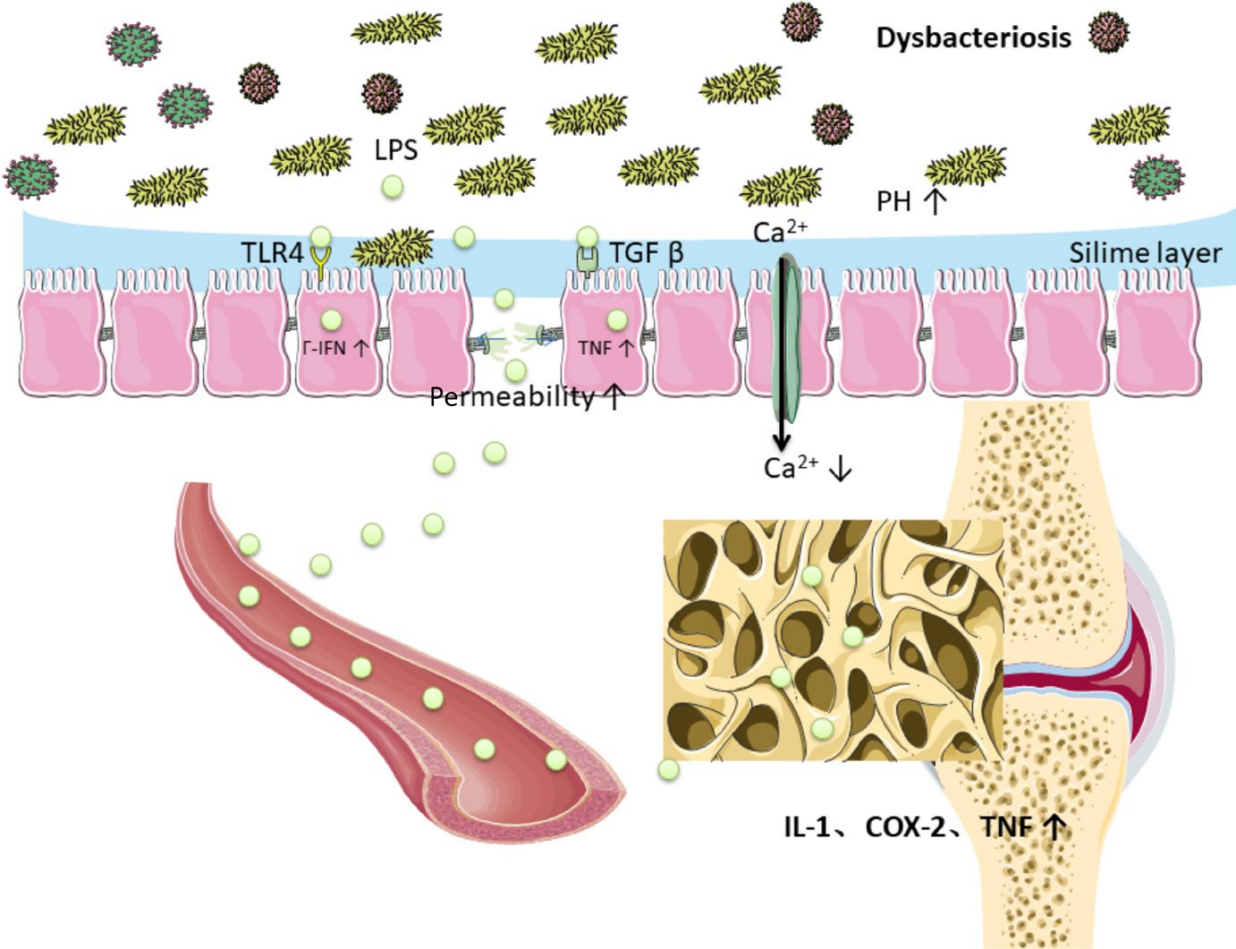
(a) Disorders of intestinal microbiota can increase the permeability of intestinal cell, which caused more LPS into the circulation system. LPS can upregulate the inflammatory mediators, interleukin (IL)‐1, cyclooxygenase (COX)‐2, and TNF in the bone metaphyseal region. (b) Disorders of intestinal microbiota can increase gut PH and decrease calcium absorption

Osteoporosis (OP) is a systemic metabolic disease characterized by low bone mass and microstructural destruction of bone tissue, which leads to an increased bone fragility and easy fracture. The diagnosis of osteoporosis has always been concerned by the medical community. Diagnostic criteria recommended by WHO depends on the lumbar 1~4 and femoral neck measured by dual‐energy X‐ray bone densitometer. It is common that the value of bone density is less than 1 standard deviation (SD) in healthy adults of the same sex and race. The drop between −1 and −2.5 SD was identified to be low bone mass. Osteoporosis occurs when the reduction was equal to and greater than −2.5 SD. The incidence of osteoporosis is increasing, which has received more and more attention from the scientific researchers. Data from the National Osteoporosis Foundation show that osteoporosis affects 10.2 million Americans over the age of 50, while 43.4 million Americans have low bone mass (Looker, Borrud, Dawson‐Hughes, Shepherd, & Wright, 2012). Moreover, the incidence of osteoporosis‐related pathological fractures was far more than strokes, heart attacks, and breast cancer each year, which increased $19B fiscal burden of the state (Burge et al., 2007). It is well known that bone mass increases from birth and peaks in adulthood. The maintenance of bone in mid‐to‐late adulthood follows different age, sex, and race related patterns, which are greatly affected by lifestyle factors (Weaver et al., 2016). The absorption of calcium and vitamin D is particularly important for the maintenance of healthy bone, and other nutrients have been gradually discovered. For example, probiotics can reduce gut PH and improve calcium absorption (Wallace, Marzorati, Spence, Weaver, & Williamson, 2017) (Figure 1). During growth, fructose oligosaccharide, galactose, soluble corn fiber (SCF), and other probiotics can increase calcium uptake in humans. The pathogenesis of osteoporosis is complex. Bone mass density is determined by bone absorption and bone formation. During a person's life, bone remodeling continues, among which there are many influencing factors. When bone formation is dominant, it is characterized by anabolism, otherwise it's catabolism. Hormonal environment, immune system, and metabolic pathways can affect this balance, and the gut microbiota could affect these pathways (Charles, Ermann, & Aliprantis, 2015).

Trillions of microbes live in the gut, evolving with their hosts together and forming mutually beneficial relationships (Backhed et al., 2012). These symbiotic microbes can be seen as a multicellular organ that interacts and influences host in a variety of ways (Clarke et al., 2014; Yano et al., 2015). Many studies have elucidated the role of intestinal microbiota in shaping the host immune system (Hooper, Dan, & Macpherson, 2012) as well as in host metabolism (Nieuwdorp, Gilijamse, Pai, & Kaplan, 2014). The composition of intestinal microbiota varies from individual to individual, while the involved functional genes are similar, which indicates that intestinal microbiota is an indivisible community to maintain the homeostasis of host (Methé et al., 2012). Probiotics are the active microorganisms that are beneficial to the host. Compared with ovariectomized mice without probiotic intervention, the bone mass of cortical bone was significantly increased in estrogen‐deficient mouse model which was fed with *Lactobacillus reuteri*. Probiotics can inhibit the activity of osteoclasts and reduce the expression level of inflammatory factors. Besides, it can promote the absorption of bone calcium and significantly increase the expression of osteogenic markers (Resta‐Lenert & Barrett, 2006). The natural prebiotics are mainly high fiber foods, including vegetables, fruits, and grains. Fructo‐oligosaccharides, inulin‐type prebiotics, can increase the amount of “beneficial” bacteria in the gut and promote the release of organic acids by stimulating the activity of bacteria, thereby lowering the pH of the intestines. Hence, prebiotics can promote the absorption of minerals and increase bone mineralization (María Isabel, Alicia, & López, 2014).

Taken together, intestinal microbiota may play a role in bone metabolism *via* influencing the host metabolism, immunity, and endocrine environment. Here, we searched relevant literature on Pubmed and then reviewed the effect of intestinal microbiota on bone metabolism through these three aspects.

## 2 EFFECTS OF GUT MICROBIOTA ON HOST METABOLISM AND BONE HOMEOSTASIS

Diet can change the species of intestinal microbiota. High‐fiber diet and fructo‐oligosaccharides can increase the number of Bifidobacteria species (David, Maurice, & Carmody, 2014). Accumulating evidence suggests that probiotics can affect host metabolism, which may protect gut epithelial cell and maintain the integrity of mucous layer. By contrasting germ‐free (GF) mice with conventionally fed mice, one previous study proved that intestinal microbiota impacts the host metabolism to influence bone density. GF mice obtained a 50% increase in femur trabecular bone volume and cortical bone (Sjogren et al., 2012). The intestinal microbiota can affect the host metabolism through a variety of ways to influence bone turnover. Here, we analyze several aspects of this field.

### 2.1 Lipopolysaccharide (LPS)

In cross‐sectional studies, high systemic LPS and LPS binding proteins were associated with low level of chronic inflammation leading to type 2 diabetes mellitus (T2DM), metabolic syndrome, and obesity (Cani et al., 2007, 2007; Creely et al., 2007; Jayashree et al., 2014; Sun et al., 2010). The bacterial cell wall composition of LPS and endotoxin is found mainly in gram‐negative bacteria, which stimulate inflammation by activating transformed growth factor (TGF) and toll‐like receptors 4 (Manco, Putignani, & Bottazzo, 2010). Characteristics of Dysbacteriosis in diabetes are reducing gram‐positive bacteria which lacks LPS. Increasing gram‐negative opportunistic pathogens, such as bacteroidetes and proteus species containing LPS are other characteristics in diabetes (Qin et al., 2012). Intestinal microbiota regulated cells permeability by sustaining the healthy and tight junctions of intestinal cells, and maintaining protective slime layer. Disorders of intestinal microbiota can increase the permeability of intestinal cell, which caused more LPS into the system of circulation, leading to metabolic dysfunction and inflammation (Horton, Wright, Smith, Hinton, & Robertson, 2013). There are many data in animal studies in this field, (Brun et al., 2007; Cani et al., 2007; Ghoshal, Witta, Zhong, De, & Eckhardt, 2009) more studies are needed in human. LPS also plays an important role in bone metabolism. Three doses of LPS time‐release pellet were implanted in 3‐month‐old rats to mimic a vivo model of chronic inflammation. In both LPS‐treated groups, bone loss occurred in their femur, suggesting LPS may reduce bone mineral density. In the high‐dose LPS group, microcomputed tomography indicated that trabecular bone volume of the proximal tibial metaphysis tended to be decreased. Furthermore, upregulation of the inflammatory mediators, interleukin (IL)‐1, cyclooxygenase (COX)‐2, and TNF was tested in the metaphyseal region (Smith et al., 2006) (Figure 1). Chongwatpol P. et al. illuminated that LPS substantially decreased trabecular bone volume, lumbar vertebra bone mineral density, and the number of the vertebral body compared with both zinc inadequate and adequate without LPS groups (Chongwatpol et al., 2015).

### 2.2 Bile acid

**Figure 2:**
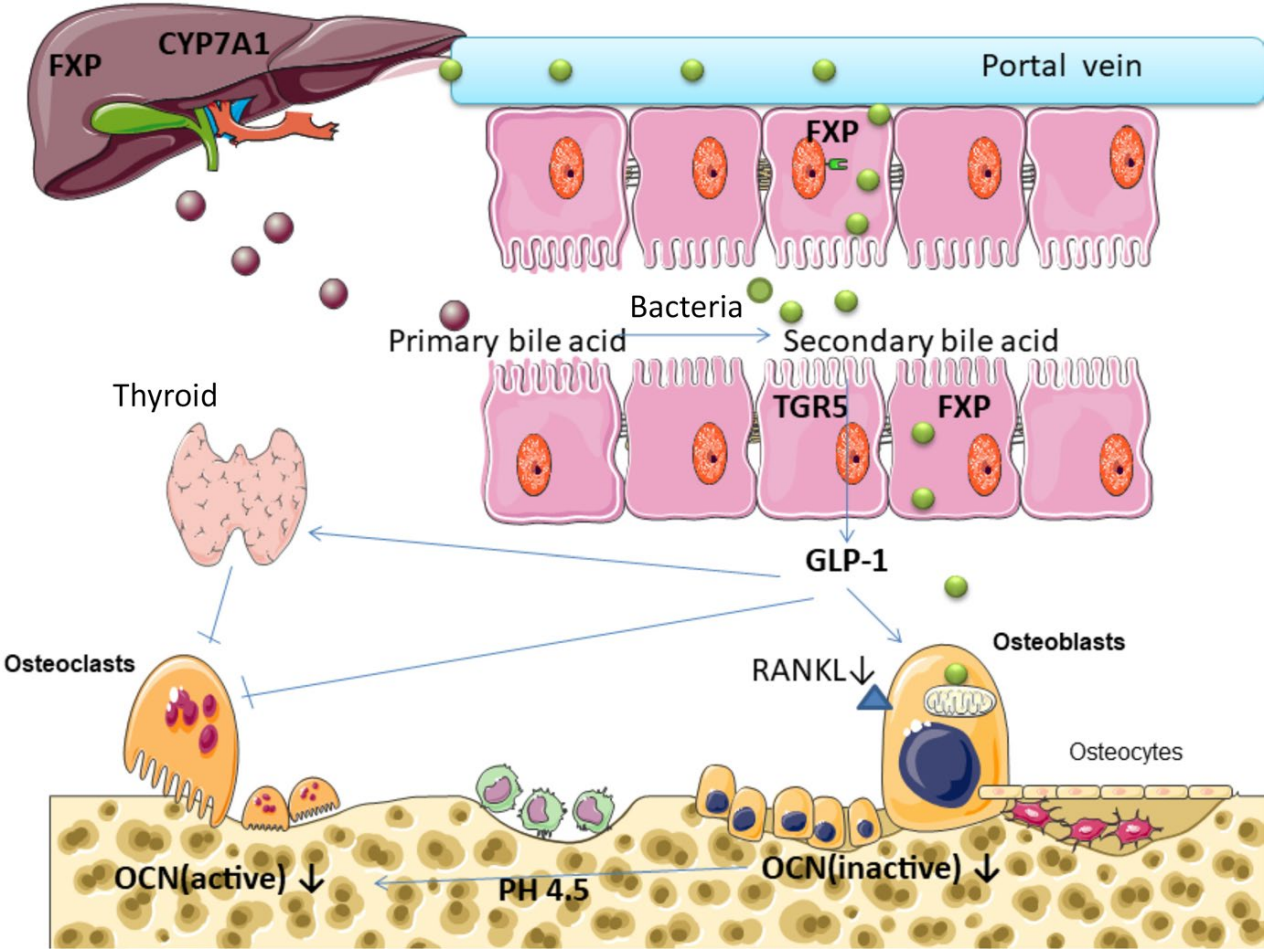
(a) Bile acids experienced a “gut‐liver axis” and were transformed to the secondary bile acid under the influence of anaerobic bacteria. (b) Through FXP and TGR5 signaling, intestinal microbial components can change the amount and the type of secondary bile acid. (c) Some types of secondary bile acid are agonists of the membrane‐bound G‐protein‐coupled receptor (TGR5). Stimulation of TGR5 can increase the production of glucagon‐like peptide‐1 (GLP‐1), a kind of enterogenous hormone, which can active thyroid C cells proliferation and promote the secretion of calcitonin, thus inhibiting bone resorption. GLP‐1 also can stimulate the proliferation of osteoblast and inhibit osteoclast.(Sandoval & D'Alessio, 2015) (d) LCA can damage osteoblasts mitochondrial and reduce cell viability. As a mild VDR ligand, LCA can reduce the gene expression of osteocalcin and RANKL

Primary bile acids were conjugated with taurine or glycine to make up bile salts in liver, then they were secreted into the small intestine. Ninety‐five percent of bile salts are transported back to the liver and enter “gut‐liver axis” when reaching the ileum. Roughly 400~600 mg of bile salts reach the large intestine (Figure 2). They experience a variety of anaerobic bacterial biological transformation to secondary bile acids, primarily lithodeoxycholic acid and deoxycholic acid (Donerner, Takamine, Lavoie, Mallonee, & Hylemon, 1997; Wells & Hylemon, 2000). The gut microbiome plays a significant role in the bile acid metabolism (Ridlon, Kang, Hylemon, & Bajaj, 2014). Through farnesoid X receptor (FXR) and G protein‐coupled bile acid receptor 5 (TGR5) signaling, intestinal microbial components can change the amount and the type of secondary bile acid, thus producing different metabolic effects (Figure 2). The enzymes contained in the deconjugation, epimerization, and dehydroxylation of bile acids are existed in many species (Labbe, Ganopolsky, Martoni, Prakash, & Jones, 2014). The variety of food can affect intestinal microbiota and the metabolism of bile acids. In mice consumed mung bean protein (MPI), the enhancement of cecum and fecal cistern was observed. These effects were eliminated in GF mice, suggesting that their effects depended on the presence of microbiota. Analysis of the 16s‐rRNA gene sequence had revealed that intake of MPI can also cause drastic changes in intestinal microbiota, such as the reduction of firmicutes and the expansion of phylum bacteroidetes (Nakatani et al., 2018). In addition, oral administration of vancomycin can reduce secondary bile acid and worse insulin sensitivity by changing intestinal microbiota (Vrieze et al., 2014). It is worth mentioning that monohydroxylated secondary lithocholic acid (LCA) can be as a kind of vitamin D receptor (VDR) ligand synthesized primarily by 7‐dehydroxylation of intestinal bacteria (Adachi et al., 2005).

It is reported that 1,25‐dihydroxyvitamin D3 played an important role in bone integrity (van Leeuwen, Van, Gj, & Pols, 2001) and mineral balance (Sutton & Macdonald, 2003). The biological effects of vitamin D are regulated by its receptor, the VDR, which is one of the steroid receptors that can control the biological effects of multiple hormones. Vitamin D also regulates genes coding for osteoprotein, (Margolis & Christakos, 2010; Shen & Christakos, 2005) osteocalcin (Ozono, Liao, Kerner, Scott, & Pike, 1990), and the receptor activator of NF‐kB ligand (RANKL), (Kim, Yamazaki, Zella, Shevde, & Pike, 2006; Kitazawa, Mori, Yamaguchi, Kondo, & Kitazawa, 2008) which directly controls the bone turnover. Due to the toxicity of LCA in osteoblasts and the ability of binding to VDR, it seems that LCA may affect bone metabolism (Figure 2). Excessive LCA may play a role in the pathogenesis of osteoporosis. In the hamster model, LCA caused osteoblasts mitochondrial damage, which reduced cell viability, (Ceryak, Bouscarel, Malavolti, & Fromm, 1998) although the concentration is as low as 100IM (Ruiz‐Gaspa et al., 2010). In many tissues, vitamin D activates the enzyme 1a, 25‐dihydroxyvitamin D3 24‐hydroxylase (CYP24A1) through a vitamin D‐dependent pathway, regulating its own metabolism. In the condition of no effects on cell viability, LCA significantly reduced CYP24A1 expression by 72% (Kim et al., 2006). CYP24A1 contains two vitamin D reaction elements (VDREs) area. These sequences depend on vitamin D to induce the gene expression (Tashiro, Abe, Oue, Yasui, & Ryoji, 2004; Tashiro, Ishii, & Ryoji, 2007). The effect of LCA on CYP24A1 is produced by the VDREs that were located in CYP24A1 promoter region, which further indicates that LCA is a mild analog of vitamin D. In addition to the ability of LCA catabolism of vitamin D, LCA reduces the other two genes expression, such as osteocalcin, closely associated with bone formation, and RANKL which is expressed in osteoblasts and regulated osteoclast formation. LCA reduced the ability of vitamin D to activate osteocalcin and RANKL genes by 79% and 56%, respectively (Ruiz‐Gaspa et al., 2010).

### 2.3 Short‐chain fatty acids (SCFAs)

Bacteria in the colon can ferment indigestible carbohydrates into SCFAs, including acetate, propionate, and butyrate. In addition, the fermentation of amino acids by intestinal bacteria also produces SCFAs. Protein fermentation accounts for 17%–38% of SCFAs in cecum and sigmoid colon (Mccabe, Britton, & Parameswaran, 2015). For enterocytes, butyrate is a significant energy source, while propionate and acetate are mainly absorbed by liver and used as a material source for gluconeogenesis (Den et al., 2013). Acting as signaling molecules, SCFAs can activate AMP kinase and free fatty acid receptors 2 and 3 (FFAR2 and 3), termed as G‐protein‐coupled receptors 43 and 41 (Zhang et al., 2015). SCFAs can inhibit lipogenesis and stimulate fatty acid oxidation (Den et al., 2013), which can prevent from the development of nonalcoholic fatty liver disease.

**Figure 3:**
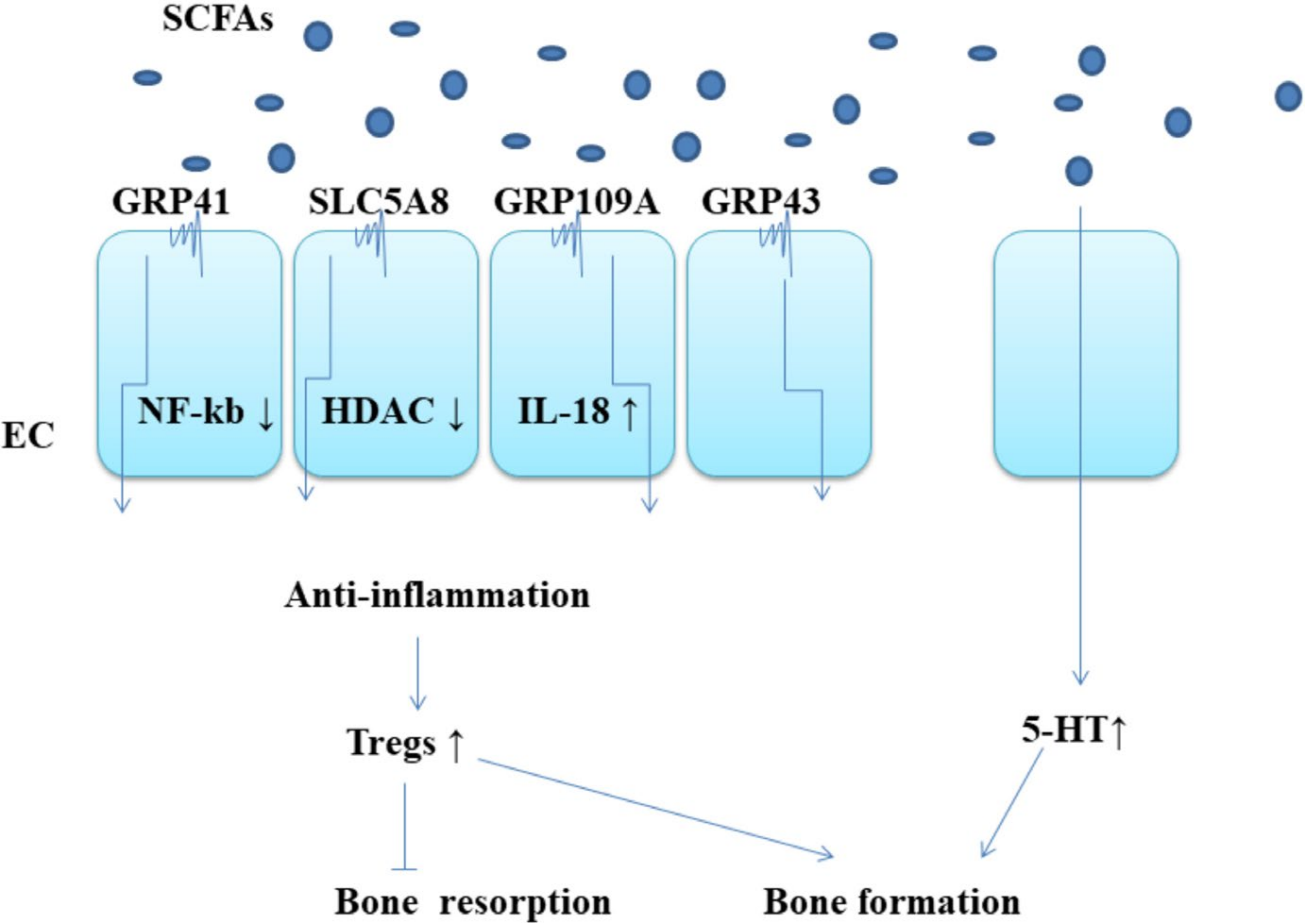
(a) SCFAs owns the ability to influence Tregs development through membrane protein (SLC5A8, GPR41, GPR43, GPR109A), which can promote an anti‐inflammatory environment. GPR109A,(Furusawa et al., 2013) activated by butyrate, suppresses intestinal inflammation by induction of IL‐18 secretion; GPR43,(Smith et al., 2013) activated by acetate promotes resolution of intestinal inflammation by inducing apoptosis of inflammatory cells; GPR41, activated(Lührs et al., 2002) by butyrate, suppresses intestinal inflammation via inhibition of NF‐kB activation; LC5A8,(Thangaraju et al., 2010) suppresses intestinal inflammation via inhibition of histone deacetylase (HDAC). (b). Enterochromaffin cells are responsible for the synthesis of 5HT that is partially modulated by GM as SCFAs increase the synthesis of 5HT.(Reigstad et al., 2015)

The oligosaccharide diet increases the production of SCFAs and changes the microbial composition, indicating the close relationship between microbiota, dietary fiber content, and SCFAs (Smiricky‐Tjardes, Grieshop, Flickinger, Bauer, & Jr, 2003). SCFAs products varied according to the types of amino acid matrix and intestinal bacteria (Dai, Jing, Wu, & Zhu, 2010; Smith & Macfarlane, 1997), such as Clostridium, Bifidobacterium, and Lactobacillus, which mainly produce SCFAs (Den et al., 2013). Bone mineral density increased significantly and briefly after low‐dose antibiotic treatment in conventionally fed mice, suggesting that bacterial microbiota may play a role in bone catabolism (Cho et al., 2012). Consistent with the study, Sjogren and his colleagues found that colonizing young adult GF animals with bacteria can lead to bone loss of trabecular and the proliferation of osteoclast (Sjogren et al., 2012). However, some other studies suggested that administration of beneficial microorganisms can moderately increase bone density (Britton et al., 2014; Mccabe et al., 2015; Mccabe, Irwin, Schaefer, & Britton, 2013; Parvaneh, Jamaluddin, Karimi, & Erfani, 2014; Weaver, 2015; Zhang et al., 2015) and prevent from bone loss during menopause (Li et al., 2016). The above inconsistent results may reflect the differences in the composition of microbiota, animal species, and gender in these studies. Bone turnover is composed of bone resorption and bone formation. When bone formation induced by bacteria is not enough to offset bone resorption, it will lead to bone catabolism. Systematic insulin‐like growth factor 1 (IGF‐1), a hormone known to have an effect on bone growth, significantly increased after microbiota transplantation. Exogenous IGF‐1 promoted the growth of longitudinal femoral (Yakar et al., 2002). Insulin‐like growth factor I receptor deletion model indicated that IGF‐1 plays a vital role in the mature growth plate and in the process of the formation of secondary ossification center (Wang et al., 2011). Compared with GF mice, circulating IGF‐1 increased in both short‐term and long‐term microbiota transplantation mice. It seems to be contrary to the result of Sjogren and his colleagues. In Sjogren's study, they colonized 3‐week‐old female GF C57Bl6/J mice with normal gut microbiota. Four weeks after colonization, the trabecular BMD had been significantly reduced compared with GF counterparts. However, in Jing's study, they colonized 2‐month‐old female GF CB6F1 mice with normal gut microbiota. Four weeks after colonization, trabecular bone mass was decreased compared with GF controls, which was the same as Sjogren's study. Furthermore, Jing also found that the serum C‐terminal telopeptides of type I collagen (CTX‐I), I N‐terminal propeptide (P1NP), and IGF‐1 were increased in colonized mice. This suggests that the bone resorption promoting effects of colonization overtake the effect on bone formation. However, 8 months after colonization, the effect on bone formation was dominant, leading to increased bone mineral density of longitudinal and radial bones (Yan et al., 2016). The duration of colonization, age, and strain of the mice may be the explanation of the contrary results in these two studies. In short‐term transplantation, IGF‐1 stimulated bone resorption dominantly, which will cause bone loss. However, IGF‐1 stimulated bone formation dominantly over a longer period, resulting in an increase of bone mass (Schwarzer et al., 2017). Antibiotic treatment reduced the level of SCFAs, IGF‐1, and P1NP. After given SCFAs, the levels of systematic IGF‐1 and bone mass in the antibiotics‐treated mice were the same as the nonantibiotic mice. Therefore, the production of SCFAs may be a mechanism by which microbial community increased the serum level of IGF‐1. Compared with mice treated with antibiotics, SCFAs supplementation increased the production of fat pad IGF‐1. Serum insulin‐like growth factor binding protein 3 (IGFBP3) was not changed. In addition, reduction of trabecular bone mass was observed after 4 months of SCFAs supplementation, similar to short‐term microbiota transplantation (Yan et al., 2016). Other mechanisms of SCFAs are shown in Figure 3.

## 3 EFFECT OF MICROBIOTA ON IMMUNE SYSTEM AND BONE HOMEOSTASIS

Recognizing that the microbiota and immune system are significant to the balance in the skeleton means that evolvement from the field of bone immunology to “osteomicrobiology,” a term coined by Ohlsson et al (Ohlsson & Sjögren, 2015). Intestinal microbiota is essential for the function and maturation of immune system. The relation between the microbiota and the skeleton was first discovered in 2012 (Sjogren et al., 2012). Compared with the control group under normal conditions, the amount of trabecular bone was increased in mice under sterile conditions. Since this phenomenon was overturn by colonization of the gut microbiota from conventionally fed mice, the evidence was compelling that results are not on account of the innate abnormalities of GF mice. This study also found that the number of CD4^+^T cells and TNF in bone marrow was lower in GF mice. In another study, probiotics seemed to increase bone density and reduce intestinal inflammation in men rather than women (Mccabe et al., 2013). The close relationship between bone loss and inflammatory conditions has long been valued by clinicians (Mbalaviele, Novack, Schett, & Teitelbaum, 2017; Redlich & Smolen, 2012). Specific strains had effects on specific immune cells, (Goto et al., 2014; Ivanov et al., 2009) and new insights have been developed on the effect of the whole microbiome on the immune system.

### 3.1 Th17 and Treg cells

T cells are a heterogeneous group of cells. By function, it can be classified into helper T cells (Th cells), inhibitory T cells (Ts cells), cytotoxic T cells (CTL or Tc cells), and delayed hypersensitivity T cells (TDTH cells).

**Figure 4:**
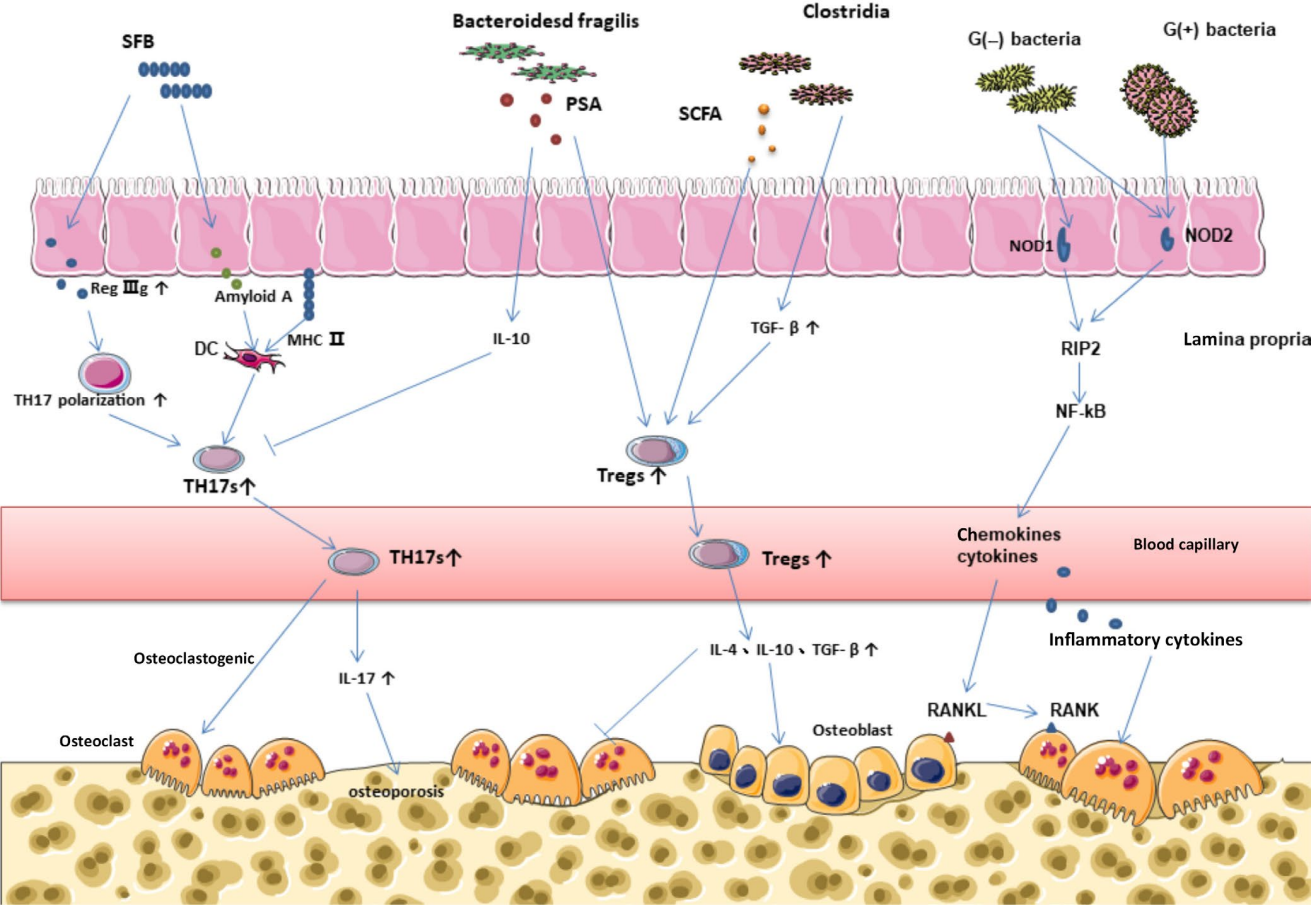
(a) Th17 cells produce a pro‐osteoclastogenic effect. SFB influenced the expression of antimicrobial proteins RegIIIg participating in Th17 cell polarization; SFB induced Th17 cell differentiation by intestinal epithelial cells production of serum amyloid A that might affect DC cytokine; Th17 cell differentiation depended on MHCII‐dependent antigen presentation of SFB antigens by intestinal dendritic cells; *Bacteroides fragilis* prevented the differentiation of Th17 cells by polysaccharide A. (b) Tregs regulated the formation of osteoclast. Treg cell differentiation was induced by short‐chain fatty acids, leading to epigenetic changes to stabilize the program; Clostridium genus provided bacterial antigens and a TGF‐b‐rich environment, resulting in the expansion of systemic Tregs. (c). NLRs bind to bacterial peptides and attract receptor interaction protein (RIP2), stimulating the NF‐kB signaling pathway, which can induce osteoclastogenesis through chemokines and cytokines

Producing interleukin‐17 (IL‐17) and many other effector cytokines, such as IL‐22, Th17 cells are important for activating innate immune mechanism, including inducing epithelial cells to produce antimicrobial peptides and recruiting neutrophils. Th17 cells play a significant role in mucosal resist against bacteria and fungi (Korn, Bettelli, Oukka, & Kuchroo, 2009). Transplantation of segmented filamentous bacteria (SFB) into GF mice increased the number of Th17 cells and mildly increased Th1 cells.(Goto et al., 2014; Ivanov et al., 2009) SFB seems to penetrate the mucous layer of the terminal ileum, contact with epithelial cells, and induce actin aggregation, possibly leading to Th17 polarization signals in the lamina propria. Little is known that Th17 polarizing signaling pathways can be initiated by SFB. It is likely that SFB affects the expression of antimicrobials proteins RegIIIg and molecules in epithelial cell, which are involved in Th17 cell polarization. In addition, studies have shown that MHCII‐dependent antigen presentation of SFB antigens by intestinal dendritic cells (DCs) is crucial for Th17 cell induction (Goto et al., 2014) (Figure 4).

CD4^+^FOXP3^+^Treg cells are stable in the intestinal mucosa and have an effect on the intestinal and systemic immune system. In GF mice, Treg cells and IL‐10 were significantly reduced (Atarashi et al., 2011; Geuking et al., 2011). In a study, researchers separated 17 strains of bacteria, which can enhance the expansion of Treg cells and induce significant anti‐inflammatory molecules, such as IL‐10 and inducible T‐cell stimulator (ICOS). These 17 strains provided a TGF‐β rich environment and bacterial antigen, thus inducing the expansion and differentiation of Treg cells (Figure 4). Genomic sequencing showed that the 17 strains belonged to IV, XIVa, and XVIII of Clostridia. The Clostridias were lack of virulence factors and significant toxins (Oshima, 2013).

Transplantation of the GF mice with IV and XIVa of Clostridia, separated from mouse droppings, resulted in an increase in systemic and the lamina propria Treg cells. Compared with thymus Tregs, the peripheral Tregs have a phenotypic characteristic, namely they react to TGF‐β and retinoic acid (Atarashi et al., 2011). Furthermore, the induction effect of intestinal microbiota on systemic Th17 and Treg cells was also confirmed (Atarashi et al., 2011; Berer et al., 2011; Yun & Gordon, 2011). Immune function was regulated by bacterial metabolites, mainly short‐chain fatty acids (SCFAs). It has been reported that butyrate exerts immunomodulatory effect on intestinal macrophages, induces Treg cell differentiation (Furusawa et al., 2013) (Figure 3).

Th17 cells are essential for estrogen‐deficient bone loss. Th17 cells are a subset of CD4^+^T cells, which can produce a pro‐osteoclastogenesis effect. In women, the increase of serum IL‐17 is closely related to osteoporosis (Molnar, Bohaty, & Somogyine‐Vari, 2014; Molnár, Bohaty, & Somogyiné‐Vári, 2014; Zhang et al., 2014). The elimination of IL17 (Deselm et al., 2012) or the use of anti‐IL17 antibody (Tyagi et al., 2014) may prevent from bone loss (Figure 4). Treg cells deficiency and inactivation are associated with some chronic inflammatory diseases. Tregs regulate the formation of osteoclast by secretion of IL‐4, IL‐10, and TGF‐β (Kelchtermans et al., 2009; Kim et al., 2007; Luo, Wang, Sun, & Li, 2011; Zaiss et al., 2007) (Figure 4) and blocking bone resorption (Yuan et al., 2010). Importantly, it is well known that estrogen directly increases the relative number of Tregs, (Tai et al., 2008) which prevents from ovx‐induced bone loss (Zaiss et al., 2010).

### 3.2 NOD1 and NOD2 signaling

The innate immune system can recognize a variety of pathogen, which is the body's first defense against extraneous pathogenic microorganisms. It recognizes pathogens through specific pattern recognition receptors (PRRs), including the nod‐like receptor family (NLR) in the cytoplasm. On the cell surface, innate immune system in the intestines identified bacteria by PRRs, such as toll‐like receptor (TLR) family and other signaling pathways. Most of the TLR signals are adjusted through MYD88 protein to stimulate the mitogen‐activated protein (MAP) kinase and proinflammatory signals of NF‐kB (Kufer & Sansonetti, 2007). In the cytoplasm, bacterial detection is carried out by NLRs, NOD1, and NOD2. They bind to bacterial peptides and attract a common protein kinase, receptor‐interaction protein (RIP2) which stimulates the NF‐kB signaling pathway, leading to gene expression of chemokines and cytokines.

NOD1 was found in many types of cells. The proinflammatory signal was induced by recognition of peptidoglycan mainly found in gram‐negative bacteria (Clarke et al., 2010). NOD2 was widely expressed in nonhematopoietic cells, bone marrow derived cells, and lymphocytes (Nigro, Rossi, Commere, Jay, & Sansonetti, 2014; Ogura et al., 2001). NOD2 can bind to all types of peptidoglycan that is found in gram‐positive and gram‐negative bacteria, inducing inflammatory response by activation of NLRs (Figure 4). Compared with conventionally fed Myd88‐/‐ mice, the cortical bone mass of GF Myd88‐/‐ mice increased significantly, indicating that the influence of GF mice on bone was independent of Myd88 protein. In contrast, the cortical bone mass of GF mice that specifically deactivated NOD1 or NOD2 did not show a significant increase, indicating that the influence of GF mice on cortical bone mineral density depended on the signals of NOD1 or NOD2 (Ohlsson et al., 2017). The effect of NOD2 on bone resorption was verified in microbiota‐induced periodontitis model. In NOD2‐deficient mice, bone resorption and the number of osteoclast were significantly reduced (Prates et al., 2014; Souza et al., 2016). Additionally, bone marrow macrophage that extracted from NOD2‐defective mice would form less osteoclast cells than that from wild‐type mice, which suggested that bone resorption induced by bacteria was relied on NOD2 signal. Furthermore, NOD2 ligand could induce osteoclastogenesis when the RANKL gene expression increased in osteoblasts.(Yang et al., 2005)

### 3.3 Wnt signaling

Wnt signaling pathway widely exists in invertebrates and vertebrates. It is a highly conservative signaling pathway in the evolution of species. Wnt signaling plays a vital role in the early development of animal embryos, organ formation, tissue regeneration, and other physiological processes. Wnt/β‐catenin signaling can be activated by some bacteria such as the *Fusobacterium nucleatum* and *Bacteroides fragilis* (Rubinstein et al., 2013; Wu, Morin, Maouyo, & Sears, 2003). Intestinal microbiota can polarize colon macrophages into M1 state, thus producing endogenous inflammatory cytokines (Yang, Wang, Moore, Lightfoot, & Huycke, 2012; Yang et al., 2013). These events were called microbial‐induced bystander effects (MIBE). TNF contributes to MIBE and activated Wnt/β‐catenin signaling (Oguma et al., 2008). One study has shown that TNF activated Wnt/β‐catenin by inducing Wnt3 and inhibiting Wnt inhibitory factor 1(WIF1). In normal colonic epithelial cells, the basal Wnt level is restricted by WIF1, which was phosphorylated and degraded continuously by protease ubiquitination (Wang, Yang, & Huycke, 2017). β‐Catenin is a key component of the Wnt/β‐catenin signaling pathway, which regulates Wnt target gene transcription when activated by various Wnt ligands (wnt10b, wnt1, wnt3, etc.). Osteoblast function was regulated by Wnt/β‐catenin signaling in almost all aspects from infancy to maturity (Hill, Später, Taketo, Birchmeier, & Hartmann, 2005; Song et al., 2012). Therefore, β‐catenin is the key target to explore the function of this pathway on osteoblast. A large number of mouse models have shown that from immature (Day, Guo, Garrett‐Beal, & Yang, 2005) to mature stage, (Kramer et al., 2010) the depletion of β‐catenin can inhibit the differentiation of osteoblasts and increase the differentiation of osteoclasts, resulting in decreasing bone mass. Wnt binds to the frizzled protein (Fz) receptor on the surface of osteoblasts, resulting in stabilization of intracellular β‐catenin. When associated with the T‐cell factor/lymphoid enhancer factor (LEF/TCF) transcription factors, β‐catenin activates the transcription of osteoprotegerin (OPG) in osteoblasts, thereby reducing bone absorption (Nd et al., 2005). As previously shown, the loss of β‐catenin leads to a significant reduction in bone mass as a result of decreased bone formation and increased bone resorption (Holmen et al., 2005).

## 4 EFFECT OF MICROBIOTA ON HORMONE AND BONE HOMEOSTASIS

Since Lyt and his colleagues observed that stress‐induced neuroendocrine hormones affect the growth of bacteria, the relationship between microbes and endocrine has been concerned (Cooper, Knowles, Brown, Mcguirl, & Dooley, 1992).

So far, it has been found that intestinal microbiota is closely related to a large number of hormone secretion, such as gut microbiota produced serotonin, (Freestone & Lyte, 2008) Lactobacillus produced gamma‐aminobutyric acid (GABA), low level of corticosterone in probiotics‐treated mice, (Asano et al., 2012) decreased levels of insulin, (Karlsson et al., 2013) low glucagon‐like peptide‐1 (GLP‐1) levels in antibiotic‐treated mice, (Wichmann et al., 2013), and probiotics (Storelli et al., 2011). As is known to all, GLP‐1 plays an important role in bone turnover. Similarly, intestinal microbiota also plays a significant role in bone turnover through many other hormones.

### 4.1 Sex hormones

There are many conflicting results in studies on the association between intestinal microbiota and sex hormones in healthy people (Arumugam et al., 2014; Ding & Schloss, 2014; Schnorr et al., 2014). In males, Ruminococcus, Bacteroides, Eubacterium, and Blautia were in a greater abundance, while Treponema in females. However, these differences may be on account of the specific way of life and cultural factors related to gender, rather than sex hormones. It is reported that intestinal microbiota may affect the balance of steroids. Certain species have the ability to metabolize sex hormones and affect their activity (Lombardi, Goldin, Boutin, & Gorbach, 1978). For example, the intestinal symbiotic *Clostridium scindens* converted glucocorticoids into androgens with hydroxysteroid hydrolase and other enzymes (Ridlon et al., 2013). *Slackia* sp. is a common member of the intestinal microbiota, which can influence the production of estrogen. The effect of intestinal microbiota on sex hormones conversion has been established.

Furthermore, numerous studies have confirmed the role of estrogen in bone metabolism. The estrogen receptors (ERs) are expressed in osteocytes, (Tomkinson, Gevers, Wit, Reeve, & Noble, 1998) osteoblasts, (Komm et al., 1988) osteoclasts (Oursler, Osdoby, Pyfferoen, Riggs, & Spelsberg, 1991), and bone marrow stromal cells. Estrogen was able to induce the apoptosis of osteoclasts and inhibit the apoptosis of osteoblasts (Kousteni et al., 2001; Sims et al., 2003). Bone turnover cycle was activated more frequently in an estrogen deficiency environment (Erik, Langdahl, Vesterby, Rungby, & Kassem, 1999). In addition to directly affect bone cells, estrogen regulated oxidative stress and the immune system to influence bone turnover cycle. Estrogen deletion reduced the capacity of mature osteoblasts by stimulating output of proinflammatory cytokines such as TNF, IL‐7, and IL‐1 (Gilbert et al., 2000; Weitzmann, Roggia, Toraldo, Weitzmann, & Pacifici, 2002). T cells are important source of TNF in ovariectomized mice and postmenopausal women (Adeel et al., 2013). In the case of T cell‐deficient mice, ovariectomies do not induce bone loss (Gao et al., 2007). TNF plays an important role in bone loss in ovariectomized mice. The key mechanisms of TNF in stimulating bone resorption were the activation of receptor activator of NF‐kB (RANK) (Cenci et al., 2000) and the induction of Th17 cells (Sugita et al., 2012). Treatment with IL‐1 and TNF inhibitors prevented from the increase of bone resorption due to estrogen deficiency (Charatcharoenwitthaya, Khosla, Atkinson, Mccready, & Riggs, 2007). Studies have shown that sex hormone‐mediated bone metabolism not only reacted through ERs, but also through androgen receptors (ARs).(Kousteni et al., 2001) Androgen inhibits bone resorption via ARs in osteoblasts and cancellous compartment. It is essential for trabecular bone accrual during growth. Furthermore, ARs signaling takes a significant role on protecting cortical thickness and strength in aging (Jardí et al., 2018). The mechanisms by which sex hormones affect bone metabolism are greatly complex. More human studies are needed to confirm whether intestinal microbiota can influence bone metabolism directly by regulating the systematic sex hormones transformation.

### 4.2 Serotonin (5‐hydroxytryptamine, 5‐HT)

**Figure 5:**
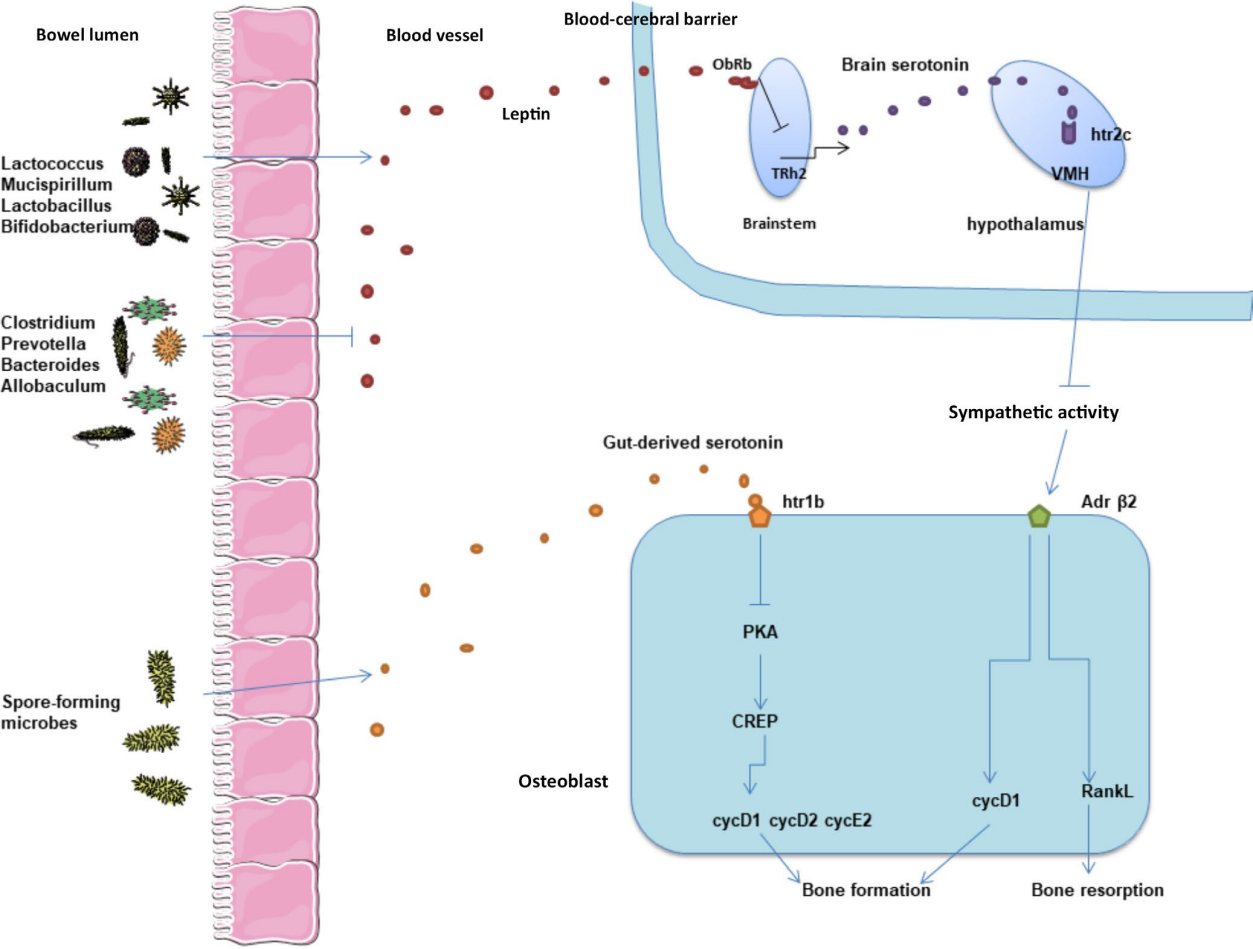
(a) Spore‐forming microbes can fully regulate serotonin levels, which regulates osteoblasts proliferation through Htr1b/PKA/CREB/cyclins signaling. (b)Specific microbiota can affect systematic level of leptin, which may influence bone mass depending on the regulation of sympathetic nervous system through brain serotonin

Serotonin, produced in the circulation, inhibits bone formation. By contrast, produced in the brain like a neurotransmitter, serotonin has a positive effect on bone mass by increasing bone formation and inhibiting bone absorption (Ducy & Karsenty, 2010) (Figure 5). 5‐HT is produced by specialized cells called enterochromaffin cells (ECs), mucosal mast cells, and intestinal muscle nerves (Gershon & Tack, 2007). More than 90% of 5‐HT are synthesized in the human gut. There are 14 different receptor subtypes in intestinal epithelial cells, (Hoffman et al., 2012) immune cells (Baganz & Blakely, 2013), and intestinal neurons.(Mawe & Hoffman, 2013) Intestinal‐derived 5‐HT regulated different functions, including intestinal movement, secretion reactions, immune response, (Baganz & Blakely, 2013) platelet aggregation, (Mercado et al., 2013) cardiac function, and bone development (Chabbiachengli et al., 2012). There are two different tryptophan hydroxylase isoenzymes Tph1 and Tph2, which regulate the synthesis of neurogenic and nonneurogenic 5‐HT. Recent studies had focused on the role of intestinal microbiota in regulating the blood levels of 5‐HT. Studies have proved that corynebacterium, streptococcus, and *E.coli* may produce 5‐HT in culture (Roshchina, 2016). The serum concentration of 5‐HT in GF mice was significantly lower than that in normal mice (Sjogren et al., 2012). Interestingly, some bacteria in healthy mice and humans can regulate the serum level of 5‐HT (Tsavkelova, Siu, Cherdyntseva, & Netrusov, 2006). Spore‐forming microbes (Sp) can fully regulate the levels of 5‐HT in serum, colon, and fecal (Figure 5). It is found that the expression level of Tph1 (the rate‐limiting enzyme for 5‐HT biosynthesis) was greatly reduced in the colon of GF mice, but the 5‐HT packaging, release, and catabolic enzymes had no difference (Yano et al., 2015). The 5‐HT transporter SLC6A4 was also highly expressed in the colon of GF mice, and was widely synthesized by intestinal cells to achieve 5‐HT absorption. This may reflect the host's compensatory response to lack of 5‐HT (Wade et al., 1996). In GF mice, transplantation of intestinal microbiota can restore the levels of 5‐HT in serum and colon at every age. At early stage, the effect of intestinal microbiota transplantation was more obvious (Yano et al., 2015). The level of 5‐HT was consistent with that of GF mice after separate transplantation of SFB or fragile bacteroidetes. However, transplantation of Sp microbes into GF mice completely restored serum and colon 5‐HT levels (Stefka et al., 2014). It is interesting that the level of 5‐HT in GF mice was also restored after transplanted with Sp microbes from healthy human, suggesting that the adjustment role of Sp microbes in 5 ‐ HT level can transfer between human and mice.(Yano et al., 2015)

There are three serotonin receptors expressed in osteoblasts, Htr1b, Htr2a, and Htr2b. Inhibition of Htr2b activity can reduce bone formation leading to a decreased bone density in female mice (Collet et al., 2008). The combination of serotonin with Htr1b on the surface of osteoblasts inhibited the production of cAMP and the phosphorylation of PKA‐mediated cAMP reaction element (CREB), which result in a decreased expression of cyclin genes and a reduced osteoblast proliferation. Similarly, osteoblast specific inhibition of CREB can lead to low bone formation phenotype and low bone mass. In Htr1b‐/‐ mice, the downregulated level of CREB normalize their high bone mass phenotype. In addition, in vitro and vivo gene expression analysis confirmed that cell cycle proteins D1, D2, and E1 were the transcription genes for CREB under the regulation of gut‐derived serotonin (Figure 5). Thus, these phenomena suggested that the direct targets of gut‐derived serotonin are osteoblasts, while Htr1b/PKA/CREB/cyclins signaling regulates its proliferation (Yadav et al., 2008).

### 4.3 Leptin

Leptin is a kind of hormone derived from dipocyte specific to vertebrate. It can regulate physiological processes such as bone mass, energy expenditure, and appetite. The long branch of vagus nerve regulates the interaction between brain and gut microbiota, known as the “gut‐brain axis”. Some evidences link microbiota to the level of leptin. First of all, use of vancomycin can cause a sharp decrease in leptin levels in rats (Lam et al., 2012). Secondly, a large number of bacteria species (such as Lactococcus, Mucispirillum, Lactobacillus, and Bifidobacterium) are positively correlated with peripheral leptin concentrations, while other bacteria species (such as Clostridium, Prevotella, Bacteroides, and Allobaculum) are negatively correlated with leptin levels (Queipoortuño et al., 2013) (Figure 5). Other evidence suggested that *L.plantarum* inhibited leptin by reducing the size of fat cells in white adipose tissue (Takemura, Okubo, & Sonoyama, 2010). The use of probiotics in a group of smokers reduced their leptin levels (Naruszewicz, Johansson, Zapolskadownar, & Bukowska, 2002).

Leptin receptor (ObRb) is expressed in the same brainstem neuron of the serotonin producing raphe nucleus (Scott et al., 2009). The combination of leptin and ObRb inhibited the expression of Tph2 gene and decreased the serotonin release of synthetic brain stem neurons (Charnay et al., 2000). Compared with mice deleting ObRb in the arcuate (ARC) and ventral hypothalamus (VMH), mice deleting ObRb serotonergic neurons showed high bone mass phenotypes (Yadav et al., 2009). Serotonin receptors Htr2c are expressed on VMH nucleus. The loss of Htr2c receptors in these neurons results in severe bone loss. This phenomenon was caused by the downregulation of bone formation and upregulation of bone resorption related to an increased sympathetic nerve activity (Yadav et al., 2009) (Figure 5). Brain serotonin plays an important role in VMH neurons through Htr2c to reduce sympathetic activity and contribute to the beneficial effect on bone mass. Leptin‐dependent central regulation of bone mass depends on the regulation of sympathetic nervous system through brain serotonin (Karsenty, 2006).

In conclusion, intestinal microbiota components can regulate bone metabolism *via* influencing the host metabolism, immunity, and endocrine environment, which may provide new ideas and targets for the clinical treatment of osteoporosis. However, most of findings need to be further validated in human studies as they are mainly drawn from animal studies.

## CONFLICT OF INTEREST

The authors have declared that no competing interests exist.

## AUTHORS CONTRIBUTION

Lishan Li (First Author) and Shitao Rao (Co‐first author) conceptualized the manuscript and wrote the first draft. Yanzhen Cheng, Xiaoyun Zhuo, Caihong Deng, Ningning Xu, Hua Zhang and Li Yang contributed to the conception of the manuscript. Hua Zhang and Li Yang thoroughly reviewed the manuscript. All authors approved the final version of the manuscript.

## ETHICS STATEMENT

None required.

## Data Availability

None required. All figures used in this review article are original.

## References

[mbo3810-bib-0001] Adachi, R. , Honma, Y. , Masuno, H. , Kawana, K. , Shimomura, L. , Yamada, S. , & Makishima, M. (2005). Selective activation of vitamin D receptor by lithocholic acid acetate, a bile acid derivative, The Journal of Lipid Research, 46, 46–57. 10.1194/jlr.M400294-JLR200 15489543

[mbo3810-bib-0002] Adeel, S. , Singh, K. , Vydareny, K. H. , Kumari, M. , Shah, E. , Weitzmann, M. N. , & Tangpricha, V. (2013). Bone loss in surgically ovariectomized premenopausal women is associated with T lymphocyte activation and thymic hypertrophy. Journal of Investigative Medicine, 61, 1178–1183. 10.2310/JIM.0000000000000016 24141238 PMC3918442

[mbo3810-bib-0003] Arumugam, M. , Raes, J. , Pelletier, E. , Le, P. D. , Yamada, T. , Mende, D. R. , … Batto, J. M. (2014). Enterotypes of the human gut microbiome. Nature, 473, 174–180. 10.1038/nature09944 PMC3728647 21508958

[mbo3810-bib-0004] Asano, Y. , Hiramoto, T. , Nishino, R. , Aiba, Y. , Kimura, T. , Yoshihara, K. , … Sudo, N. (2012). Critical role of gut microbiota in the production of biologically active, free catecholamines in the gut lumen of mice. American Journal of Physiology Gastrointestinal & Liver Physiology, 303, G1288–G1295. 10.1152/ajpgi.00341.2012 23064760

[mbo3810-bib-0005] Atarashi, K. , Tanoue, T. , Shima, T. , Imaoka, A. , Kuwahara, T. , Momose, Y. , … Ohba, Y. (2011). Induction of colonic regulatory T cells by indigenous clostridium species. Science, 331, 337–341. 10.1126/science.1198469 21205640 PMC3969237

[mbo3810-bib-0006] Backhed, F. , Fraser, C. M. , Ringel, Y. , Sanders, M. E. , Sartor, R. B. , Sherman, P. M. , … Finlay, B. B. (2012). Defining a healthy human gut microbiome: Current concepts, future directions, and clinical applications. Cell Host & Microbe, 12, 611–622. 10.1016/j.chom.2012.10.012 23159051

[mbo3810-bib-0007] Baganz, N. L. , & Blakely, R. D. (2013). A dialogue between the immune system and brain, spoken in the language of serotonin. Acs Chemical Neuroscience, 4, 48–63. 10.1021/cn300186b 23336044 PMC3547518

[mbo3810-bib-0008] Berer, K. , Mues, M. , Koutrolos, M. , Rasbi, Z. A. , Boziki, M. , Johner, C. , … Krishnamoorthy, G. (2011). Commensal microbiota and myelin autoantigen cooperate to trigger autoimmune demyelination. Nature, 479, 538–542. 10.1038/nature10554 22031325

[mbo3810-bib-0009] Britton, R. A. , Irwin, R. , Quach, D. , Schaefer, L. , Zhang, J. , Lee, T. , … Mccabe, L. R. (2014). Probiotic L. reuteri treatment prevents bone loss in a menopausal ovariectomized mouse model. Journal of Cellular Physiology, 229, 1822–1830. 10.1002/jcp.24636 24677054 PMC4129456

[mbo3810-bib-0010] Brun, P. , Castagliuolo, I. , Leo, V. D. , Buda, A. , Pinzani, M. , Palù, G. , & Martines, D. (2007). Increased intestinal permeability in obese mice: New evidence in the pathogenesis of nonalcoholic steatohepatitis. American Journal of Physiology. Gastrointestinal and Liver Physiology, 292, G518 10.1152/ajpgi.00024.2006 17023554

[mbo3810-bib-0011] Burge, R. , Dawson‐Hughes, B. , Solomon, D. H. , Wong, J. B. , King, A. , & Tosteson, A. (2007). Incidence and economic burden of osteoporosis‐related fractures in the United States, 2005‐2025. Journal of Bone & Mineral Research, 22, 465–475. 10.1359/jbmr.061113 17144789

[mbo3810-bib-0012] Cani, P. D. , Amar, J. , Iglesias, M. A. , Poggi, M. , Knauf, C. , Bastelica, D. , … Chabo, C. (2007). Metabolic endotoxemia initiates obesity and insulin resistance. Diabetes, 56, 1761 10.2337/db06-1491 17456850

[mbo3810-bib-0013] Cani, P. D. , Neyrinck, A. M. , Fava, F. , Knauf, C. , Burcelin, R. G. , Tuohy, K. M. , … Delzenne, N. M. (2007). Selective increases of bifidobacteria in gut microflora improve high‐fat‐diet‐induced diabetes in mice through a mechanism associated with endotoxaemia. Diabetologia, 50, 2374–2383. 10.1007/s00125-007-0791-0 17823788

[mbo3810-bib-0014] Cenci, S. , Weitzmann, M. N. , Roggia, C. , Namba, N. , Novack, D. , Woodring, J. , & Pacifici, R. (2000). Estrogen deficiency induces bone loss by enhancing T‐cell production of TNF‐α. Journal of Clinical Investigation, 106, 1229–1237. 10.1172/JCI11066 11086024 PMC381439

[mbo3810-bib-0015] Ceryak, S. , Bouscarel, B. , Malavolti, M. , & Fromm, H. (1998). Extrahepatic deposition and cytotoxicity of lithocholic acid: Studies in two hamster models of hepatic failure and in cultured human fibroblasts. Hepatology, 27, 546–556. 10.1002/(ISSN)1527-3350 9462656

[mbo3810-bib-0016] Chabbiachengli, Y. , Coudert, A. E. , Callebert, J. , Geoffroy, V. , Côté, F. , Collet, C. , & de Vernejoul, M. C. (2012). Decreased osteoclastogenesis in serotonin‐deficient mice. Proceedings of the National Academy of Sciences of the United States of America, 109, 2567–2572. 10.1073/pnas.1117792109 22308416 PMC3289318

[mbo3810-bib-0017] Charatcharoenwitthaya, N. , Khosla, S. , Atkinson, E. J. , Mccready, L. K. , & Riggs, B. L. (2007). Effect of Blockade of TNF‐α and Interleukin‐1 Action on Bone Resorption in Early Postmenopausal Women. Journal of Bone & Mineral Research, 22, 724–729. 10.1359/jbmr.070207 17295604

[mbo3810-bib-0018] Charles, J. F. , Ermann, J. , & Aliprantis, A. O. (2015). The intestinal microbiome and skeletal fitness: Connecting bugs and bones. Clinical Immunology, 159, 163–169. 10.1016/j.clim.2015.03.019 25840106 PMC4560610

[mbo3810-bib-0019] Charnay, Y. , Cusin, I. , Vallet, P. G. , Muzzin, P. , Rohnerjeanrenaud, F. , & Bouras, C. (2000). Intracerebroventricular infusion of leptin decreases serotonin transporter binding sites in the frontal cortex of the rat. Neuroscience Letters, 283, 89–92. 10.1016/S0304-3940(00)00951-4 10739882

[mbo3810-bib-0020] Cho, I. , Yamanishi, S. , Cox, L. , Methé, B. A. , Zavadil, J. , Li, K. , … Teitler, I. (2012). Antibiotics in early life alter the murine colonic microbiome and adiposity. Nature, 488, 621–626. 10.1038/nature11400 22914093 PMC3553221

[mbo3810-bib-0021] Chongwatpol, P. , Rendina‐Ruedy, E. , Stoecker, B. J. , Clarke, S. L. , Lucas, E. A. , & Smith, B. J. (2015). Implications of compromised zinc status on bone loss associated with chronic inflammation in C57BL/6 mice. Journal of Inflammation Research, 8, 117–128. 26203271 10.2147/JIR.S82261 PMC4508086

[mbo3810-bib-0022] Clarke, T. B. , Davis, K. M. , Lysenko, E. S. , Zhou, A. Y. , Yu, Y. , & Weiser, J. N. (2010). Recognition of Peptidoglycan from the Microbiota by Nod1 Enhances Systemic Innate Immunity. Nature Medicine, 16, 228–231. 10.1038/nm.2087 PMC4497535 20081863

[mbo3810-bib-0023] Clarke, G. , Stilling, R. M. , Kennedy, P. J. , Stanton, C. , Cryan, J. F. , & Dinan, T. G. (2014). Minireview: Gut microbiota: The neglected endocrine organ. Molecular Endocrinology, 28, 1221–1238. 10.1210/me.2014-1108 24892638 PMC5414803

[mbo3810-bib-0024] Collet, C. , Schiltz, C. , Geoffroy, V. , Maroteaux, L. , Launay, J. M. , & Vernejoul, M. C. D. (2008). The serotonin 5‐HT2B receptor controls bone mass via osteoblast recruitment and proliferation. Faseb Journal, 22, 418–427. 10.1096/fj.07-9209com 17846081 PMC5409955

[mbo3810-bib-0025] Cooper, R. A. , Knowles, P. F. , Brown, D. E. , Mcguirl, M. A. , & Dooley, D. M. (1992). Evidence for copper and 3,4,6‐trihydroxyphenylalanine quinone cofactors in an amine oxidase from the gram‐negative bacterium Escherichia coli K‐12. Biochemical Journal, 288(Pt 2), 337–340. 10.1042/bj2880337 1334402 PMC1132015

[mbo3810-bib-0026] Creely, S. J. , Mcternan, P. G. , Kusminski, C. M. , Fisher, F. M. , Silva, N. F. D. , Khanolkar, M. , … Kumar, S. (2007). Lipopolysaccharide activates an innate immune system response in human adipose tissue in obesity and type 2 diabetes. American Journal of Physiology. Endocrinology and Metabolism, 292, E740–E747. 10.1152/ajpendo.00302.2006 17090751

[mbo3810-bib-0027] Dai, Z. L. , Jing, Z. , Wu, G. Y. , & Zhu, W. Y. (2010). Utilization of amino acids by bacteria from the pig small intestine. Amino Acids, 39, 1201–1215. 10.1007/s00726-010-0556-9 20300787

[mbo3810-bib-0028] David, L. A. , Maurice, C. F. , & Carmody, R. N. (2014). Diet rapidly and reproducibly alters the human gut microbiome. Nature, 505, 559–563. 10.1038/nature12820 24336217 PMC3957428

[mbo3810-bib-0029] Day, T. F. , Guo, X. , Garrett‐Beal, L. , & Yang, Y. (2005). Wnt/β‐catenin signaling in mesenchymal progenitors controls osteoblast and chondrocyte differentiation during vertebrate skeletogenesis. Developmental Cell, 8, 739–750. 10.1016/j.devcel.2005.03.016 15866164

[mbo3810-bib-0030] Den, B. G. , Van, E. K. , Groen, A. K. , Venema, K. , Reijngoud, D. J. , & Bakker, B. M. (2013). The role of short‐chain fatty acids in the interplay between diet, gut microbiota, and host energy metabolism. Journal of Lipid Research, 54, 2325–2340. 23821742 10.1194/jlr.R036012 PMC3735932

[mbo3810-bib-0031] Deselm, C. J. , Takahata, Y. , Warren, J. , Chappel, J. C. , Khan, T. , Li, X. , … Zou, W. (2012). IL‐17 mediates estrogen‐deficient osteoporosis in an Act1‐dependent manner. Journal of Cellular Biochemistry, 113, 2895–2902. 10.1002/jcb.24165 22511335 PMC3640304

[mbo3810-bib-0032] Ding, T. , & Schloss, P. D. (2014). Dynamics and associations of microbial community types across the human body. Nature, 509, 357–360. 10.1038/nature13178 24739969 PMC4139711

[mbo3810-bib-0033] Donerner, K. C. , Takamine, F. , Lavoie, C. P. , Mallonee, D. H. , & Hylemon, P. B. (1997). Assessment of fecal bacteria with bile acid 7 alpha‐dehydroxylating activity for the presence of bai‐like genes. Applied and Environment Microbiology, 63, 1185–1188. 10.1128/aem.63.3.1185-1188.1997 PMC168411 9055436

[mbo3810-bib-0034] Ducy, P. , & Karsenty, G. (2010). The two faces of serotonin in bone biology. Journal of Cell Biology, 191, 7–13. 10.1083/jcb.201006123 20921133 PMC2953449

[mbo3810-bib-0035] Erik, F. E. M. D. , Langdahl, B. , Vesterby, A. , Rungby, J. , & Kassem, M. (1999). Hormone replacement therapy prevents osteoclastic hyperactivity: A histomorphometric study in early postmenopausal women. Journal of Bone & Mineral Research, 14, 1217–1221. 10404024 10.1359/jbmr.1999.14.7.1217

[mbo3810-bib-0036] Freestone, P. P. E. , & Lyte, M. (2008). Chapter 2 microbial endocrinology: Experimental design issues in the study of interkingdom signalling in infectious disease, Advances in Applied Microbiology, 64, 75–105. 10.1016/S0065-2164(08)00402-4 18485281

[mbo3810-bib-0037] Furusawa, Y. , Obata, Y. , Fukuda, S. , Endo, T. A. , Nakato, G. , Takahashi, D. , … Kato, T. (2013). Commensal microbe‐derived butyrate induces the differentiation of colonic regulatory T cells. Nature, 504, 446–450. 10.1038/nature12721 24226770

[mbo3810-bib-0038] Gao, Y. , Grassi, F. , Ryan, M. R. , Terauchi, M. , Page, K. , Yang, X. , … Pacifici, R. (2007). IFN‐gamma stimulates osteoclast formation and bone loss in vivo via antigen‐driven T cell activation. Journal of Clinical Investigation, 117, 122–132. 10.1172/JCI30074 17173138 PMC1697800

[mbo3810-bib-0039] Gershon, M. D. , & Tack, J. (2007). The serotonin signaling system: From basic understanding to drug development for functional GI disorders. Gastroenterology, 132, 397–414. 10.1053/j.gastro.2006.11.002 17241888

[mbo3810-bib-0040] Geuking, M. B. , Cahenzli, J. , Lawson, M. A. , Ng, D. C. , Slack, E. , Hapfelmeier, S. , … Macpherson, A. J. (2011). Intestinal bacterial colonization induces mutualistic regulatory T cell responses. Gut Microbes, 34, 794–806. 10.1016/j.immuni.2011.03.021 21596591

[mbo3810-bib-0041] Ghoshal, S. , Witta, J. , Zhong, J. , De, V. W. , & Eckhardt, E. (2009). Chylomicrons promote intestinal absorption of lipopolysaccharides. Journal of Lipid Research, 50, 90 10.1194/jlr.M800156-JLR200 18815435

[mbo3810-bib-0042] Gilbert, L. , He, X. , Farmer, P. , Boden, S. , Kozlowski, M. , Rubin, J. , & Nanes, M. S. (2000). Inhibition of osteoblast differentiation by tumor necrosis factor‐alpha. Endocrinology, 141, 3956–3964. 10.1210/endo.141.11.7739 11089525

[mbo3810-bib-0043] Goto, Y. , Panea, C. , Nakato, G. , Cebula, A. , Lee, C. , Diez, M. G. , … Ivanov, I. I. (2014). Segmented filamentous bacteria antigens presented by intestinal dendritic cells drive mucosal Th17 cell differentiation. Immunity, 40, 594–607. 10.1016/j.immuni.2014.03.005 24684957 PMC4084624

[mbo3810-bib-0044] Hill, T. P. , Später, D. , Taketo, M. M. , Birchmeier, W. , & Hartmann, C. (2005). Canonical Wnt/β‐catenin signaling prevents osteoblasts from differentiating into chondrocytes. Developmental Cell, 8, 727–738. 10.1016/j.devcel.2005.02.013 15866163

[mbo3810-bib-0045] Hoffman, J. M. , Tyler, K. , Maceachern, S. J. , Balemba, O. B. , Johnson, A. C. , Brooks, E. M. , … Galligan, J. J. (2012). Activation of colonic mucosal 5‐HT(4) receptors accelerates propulsive motility and inhibits visceral hypersensitivity. Gastroenterology, 142, 844–854. 10.1053/j.gastro.2011.12.041 22226658 PMC3477545

[mbo3810-bib-0046] Holmen, S. L. , Zylstra, C. R. , Mukherjee, A. , Sigler, R. E. , Faugere, M. C. , Bouxsein, M. L. , … Williams, B. O. (2005). Essential role of beta‐catenin in postnatal bone acquisition. Journal of Biological Chemistry, 280, 21162–21168. 10.1074/jbc.M501900200 15802266

[mbo3810-bib-0047] Hooper, L. V. , Dan, R. L. , & Macpherson, A. J. (2012). Interactions between the microbiota and the immune system. Science, 336, 1268–1273. 10.1126/science.1223490 22674334 PMC4420145

[mbo3810-bib-0048] Horton, F. , Wright, J. , Smith, L. , Hinton, P. J. , & Robertson, M. D. (2013). Increased intestinal permeability to oral chromium (51 Cr) ‐ EDTA in human Type2 diabetes. Diabetic Medicine, 31, 559–563. 24236770 10.1111/dme.12360

[mbo3810-bib-0049] Ivanov, I. I. , Atarashi, K. , Manel, N. , Brodie, E. L. , Shima, T. , Karaoz, U. , … Lynch, S. V. (2009). Induction of intestinal Th17 cells by segmented filamentous bacteria. Cell, 139, 485–498. 10.1016/j.cell.2009.09.033 19836068 PMC2796826

[mbo3810-bib-0050] Jardí, F. , Kim, N. , Laurent, M. R. , Khalil, R. , Deboel, L. , Schollaert, D. , … Vanderschueren, D. (2018). Androgen receptor in neurons slows age‐related cortical thinning in male mice. Journal of Bone and Mineral Research. 10.1002/jbmr.3625 30496619

[mbo3810-bib-0051] Jayashree, B. , Bibin, Y. S. , Prabhu, D. , Shanthirani, C. S. , Gokulakrishnan, K. , Lakshmi, B. S. , … Balasubramanyam, M. (2014). Increased circulatory levels of lipopolysaccharide (LPS) and zonulin signify novel biomarkers of proinflammation in patients with type 2 diabetes. Molecular & Cellular Biochemistry, 388, 203–210. 10.1007/s11010-013-1911-4 24347174

[mbo3810-bib-0052] Karlsson, F. H. , Tremaroli, V. , Nookaew, I. , Bergström, G. , Behre, C. J. , Fagerberg, B. , … Bäckhed, F. (2013). Gut metagenome in European women with normal, impaired and diabetic glucose control. Nature, 498, 99–103. 10.1038/nature12198 23719380

[mbo3810-bib-0053] Karsenty, G. (2006). Convergence between bone and energy homeostases: Leptin regulation of bone mass. Cell Metabolism, 4, 341–348. 10.1016/j.cmet.2006.10.008 17084709

[mbo3810-bib-0054] Kelchtermans, H. , Geboes, L. , Mitera, T. , Huskens, D. , Leclercq, G. , & Matthys, P. (2009). Activated CD4 + CD25 + regulatory T cells inhibit osteoclastogenesis and collagen‐induced arthritis. Annals of the Rheumatic Diseases, 68, 744–750. 10.1136/ard.2007.086066 18480308

[mbo3810-bib-0055] Kim, Y. G. , Lee, C. K. , Nah, S. S. , Mun, S. H. , Yoo, B. , & Moon, H. B. (2007). Human CD4 + CD25 + regulatory T cells inhibit the differentiation of osteoclasts from peripheral blood mononuclear cells. Biochemical & Biophysical Research Communications, 357, 1046–1052. 10.1016/j.bbrc.2007.04.042 17462597

[mbo3810-bib-0056] Kim, S. , Yamazaki, M. , Zella, L. A. , Shevde, N. K. , & Pike, J. W. (2006). Activation of receptor activator of NF‐κB ligand gene expression by 1,25‐dihydroxyvitamin D3 Is mediated through multiple long‐range enhancers. Molecular & Cellular Biology, 26, 6469–6486. 10.1128/MCB.00353-06 16914732 PMC1592822

[mbo3810-bib-0057] Kitazawa, R. , Mori, K. , Yamaguchi, A. , Kondo, T. , & Kitazawa, S. (2008). Modulation of mouse RANKL gene expression by Runx2 and vitamin D3. Journal of Cellular Biochemistry, 105, 1289–1297. 10.1002/jcb.21929 18814144

[mbo3810-bib-0058] Komm, B. S. , Terpening, C. M. , Benz, D. J. , Graeme, K. A. , Gallegos, A. , Korc, M. , … Haussler, M. R. (1988). Estrogen binding, receptor mRNA, and biologic response in osteoblast‐like osteosarcoma cells. Science, 241, 81–84. 10.1126/science.3164526 3164526

[mbo3810-bib-0059] Korn, T. , Bettelli, E. , Oukka, M. , & Kuchroo, V. K. (2009). IL‐17 and Th17 cells. Annual Review of Immunology, 8, 485–517. 10.1146/annurev.immunol.021908.132710 19132915

[mbo3810-bib-0060] Kousteni, S. , Bellido, T. , Plotkin, L. I. , O'Brien, C. A. , Bodenner, D. L. , Han, L. , … Katzenellenbogen, B. S. (2001). Nongenotropic, sex‐nonspecific signaling through the estrogen or androgen receptors. Cell, 104, 719–730. 11257226

[mbo3810-bib-0061] Kramer, I. , Halleux, C. , Keller, H. , Pegurri, M. , Gooi, J. H. , Weber, P. B. , … Kneissel, M. (2010). Osteocyte Wnt/beta‐catenin signaling is required for normal bone homeostasis. Molecular & Cellular Biology, 30, 3071–3085. 10.1128/MCB.01428-09 20404086 PMC2876685

[mbo3810-bib-0062] Kufer, T. A. , & Sansonetti, P. J. (2007). Sensing of bacteria: NOD a lonely job. Current Opinion in Microbiology, 10, 62–69. 10.1016/j.mib.2006.11.003 17161646

[mbo3810-bib-0063] Labbe, A. , Ganopolsky, J. G. , Martoni, C. J. , Prakash, S. , & Jones, M. L. (2014). Bacterial bile metabolising gene abundance in Crohn's, ulcerative colitis and type 2 diabetes metagenomes. PLoS ONE, 9, 115–175. 10.1371/journal.pone.0115175 PMC4269443 25517115

[mbo3810-bib-0064] Lam, V. , Su, J. , Koprowski, S. , Hsu, A. , Tweddell, J. S. , Rafiee, P. , … Baker, J. E. (2012). Intestinal microbiota determine severity of myocardial infarction in rats. Faseb Journal, 26, 1727–1735. 10.1096/fj.11-197921 22247331 PMC3316900

[mbo3810-bib-0065] van Leeuwen, J. P. , Van, D. M. , Gj, V. D. B. , & Pols, H. A. (2001). Vitamin D control of osteoblast function and bone extracellular matrix mineralization. Critical Reviews in Eukaryotic Gene Expression, 11, 199–226. 11693961

[mbo3810-bib-0066] Li, J. Y. , Chassaing, B. , Tyagi, A. M. , Vaccaro, C. , Luo, T. , Adams, J. , … Gewirtz, A. T. (2016). Sex steroid deficiency–associated bone loss is microbiota dependent and prevented by probiotics. Journal of Clinical Investigation, 126, 2049–2063. 10.1172/JCI86062 27111232 PMC4887186

[mbo3810-bib-0067] Lombardi, P. , Goldin, B. , Boutin, E. , & Gorbach, S. L. (1978). Metabolism of androgens and estrogens by human fecal microorganisms. Journal of Steroid Biochemistry, 9, 795–801. 10.1016/0022-4731(78)90203-0 713557

[mbo3810-bib-0068] Looker, A. C. , Borrud, L. G. , Dawson‐Hughes, B. , Shepherd, J. A. , & Wright, N. C. (2012). Osteoporosis or low bone mass at the femur neck or lumbar spine in older adults: United States, 2005–2008. NCHS Data Brief, 93, 1–8. 22617299

[mbo3810-bib-0069] Lührs, H. , Gerke, T. , Müller, J. G. , Melcher, R. , Schauber, J. , Boxberger, F. , … Menzel, T. (2002). Butyrate inhibits NF‐ÎºB activation in lamina propria macrophages of patients with ulcerative colitis. Scandinavian Journal of Gastroenterology, 37, 458–466. 10.1080/003655202317316105 11989838

[mbo3810-bib-0070] Luo, C. Y. , Wang, L. , Sun, C. , & Li, D. J. (2011). Estrogen enhances the functions of CD4|[plus]|CD25|[plus]|Foxp3|[plus]| regulatory T cells that suppress osteoclast differentiation and bone resorption in vitro. Cellular & Molecular Immunology, 8, 50–58. 10.1038/cmi.2010.54 21200384 PMC4002989

[mbo3810-bib-0071] Manco, M. , Putignani, L. , & Bottazzo, G. F. (2010). Gut microbiota, lipopolysaccharides, and innate immunity in the pathogenesis of obesity and cardiovascular risk. Endocrine Reviews, 31, 817–844. 10.1210/er.2009-0030 20592272

[mbo3810-bib-0072] Margolis, R. N. , & Christakos, S. (2010). The nuclear receptor superfamily of steroid hormones and vitamin D gene regulation. An update. Annals of the New York Academy of Sciences, 1192, 208–214. 10.1111/j.1749-6632.2009.05227.x 20392238

[mbo3810-bib-0073] María Isabel, G. V. , Alicia, D. R. , & López, M. G. (2014). Agave fructans: Their effect on mineral absorption and bone mineral content. Journal of Medicinal Food, 17, 1247–1255. 25069021 10.1089/jmf.2013.0137

[mbo3810-bib-0074] Mawe, G. M. , & Hoffman, J. M. (2013). Serotonin signalling in the gut‐functions, dysfunctions and therapeutic targets. Nature Reviews Gastroenterology & Hepatology, 10, 473–486. 10.1038/nrgastro.2013.105 23797870 PMC4048923

[mbo3810-bib-0075] Mbalaviele, G. , Novack, D. V. , Schett, G. , & Teitelbaum, S. L. (2017). Inflammatory osteolysis: A conspiracy against bone. Journal of Clinical Investigation, 127, 2030–2039. 10.1172/JCI93356 28569732 PMC5451216

[mbo3810-bib-0076] Mccabe, L. , Britton, R. A. , & Parameswaran, N. (2015). Prebiotic and probiotic regulation of bone health: Role of the intestine and its microbiome. Current Osteoporosis Reports, 13, 363–371. 10.1007/s11914-015-0292-x 26419466 PMC4623939

[mbo3810-bib-0077] Mccabe, L. R. , Irwin, R. , Schaefer, L. , & Britton, R. A. (2013). Probiotic use decreases intestinal inflammation and increases bone density in healthy male but not female mice. Journal of Cellular Physiology, 228, 1793–1798. 10.1002/jcp.24340 23389860 PMC4091780

[mbo3810-bib-0078] Mercado, C. P. , Quintero, M. V. , Li, Y. , Singh, P. , Byrd, A. K. , Talabnin, K. , … Kuberan, B. (2013). A serotonin‐induced N‐glycan switch regulates platelet aggregation. Scientific Reports, 3, 1–9. 10.1038/srep02795 PMC3786303 24077408

[mbo3810-bib-0079] Methé, B. A. , Nelson, K. E. , Pop, M. , Creasy, H. H. , Giglio, M. G. , Huttenhower, C. , … Jonathan, H. (2012). A framework for human microbiome research. Nature, 486, 215–221. 22699610 10.1038/nature11209 PMC3377744

[mbo3810-bib-0080] Molnar, I. , Bohaty, I. , & Somogyine‐Vari, E. (2014). IL‐17A‐mediated sRANK ligand elevation involved in postmenopausal osteoporosis. Osteoporosis International, 25, 783–786. 10.1007/s00198-013-2548-6 24337660

[mbo3810-bib-0081] Molnár, I. , Bohaty, I. , & Somogyiné‐Vári, É. (2014). High prevalence of increased interleukin‐17A serum levels in postmenopausal estrogen deficiency. Menopause, 21, 749–752. 10.1097/GME.0000000000000125 24253487

[mbo3810-bib-0082] Nakatani, A. , Li, X. , Miyamoto, J. , Igarashi, M. , Watanabe, H. , Sutou, A. , … Kohno, M. (2018). Dietary mung bean protein reduces high‐fat diet‐induced weight gain by modulating host bile acid metabolism in a gut microbiota‐dependent manner. Biochemical & Biophysical Research Communications, 501, 955–961. 10.1016/j.bbrc.2018.05.090 29777704

[mbo3810-bib-0083] Naruszewicz, M. , Johansson, M. L. , Zapolskadownar, D. , & Bukowska, H. (2002). Effect of Lactobacillus plantarum 299v on cardiovascular disease risk factors in smokers. American Journal of Clinical Nutrition, 76, 1249–1255. 10.1093/ajcn/76.6.1249 12450890

[mbo3810-bib-0084] Nd, G. D. , Bialek, P. , Ahn, J. D. , Starbuck, M. , Patel, M. S. , Clevers, H. , … Lang, R. A. (2005). Canonical Wnt signaling in differentiated osteoblasts controls osteoclast differentiation. Developmental Cell, 8, 751–764. 15866165 10.1016/j.devcel.2005.02.017

[mbo3810-bib-0085] Nieuwdorp, M. , Gilijamse, P. W. , Pai, N. , & Kaplan, L. M. (2014). Role of the microbiome in energy regulation and metabolism. Gastroenterology, 146, 1525–1533. 10.1053/j.gastro.2014.02.008 24560870

[mbo3810-bib-0086] Nigro, G. , Rossi, R. , Commere, P. H. , Jay, P. , & Sansonetti, P. (2014). The cytosolic bacterial peptidoglycan sensor Nod2 affords stem cell protection and links microbes to gut epithelial regeneration. Cell Host & Microbe, 15, 792–811. 10.1016/j.chom.2014.05.003 24882705

[mbo3810-bib-0087] Oguma, K. , Oshima, H. , Aoki, M. , Uchio, R. , Naka, K. , Nakamura, S. , … Oshima, M. (2008). Activated macrophages promote Wnt signalling through tumour necrosis factor‐α in gastric tumour cells. Embo Journal, 27, 1671–1681. 10.1038/emboj.2008.105 18511911 PMC2413189

[mbo3810-bib-0088] Ogura, Y. , Inohara, N. , Benito, A. , Chen, F. F. , Yamaoka, S. , & Núñez, G. (2001). Nod2, a Nod1/Apaf‐1 family member that is restricted to monocytes and activates NF‐κB. Journal of Biological Chemistry, 276, 4812–4818. 10.1074/jbc.M008072200 11087742

[mbo3810-bib-0089] Ohlsson, C. , Nigro, G. , Boneca, I. G. , Bäckhed, F. , Sansonetti, P. , & Sjögren, K. (2017). Regulation of bone mass by the gut microbiota is dependent on NOD1 and NOD2 signaling. Cellular Immunology, 317, 55–58. 10.1016/j.cellimm.2017.05.003 28576260

[mbo3810-bib-0090] Ohlsson, C. , & Sjögren, K. (2015). Effects of the gut microbiota on bone mass. Trends in Endocrinology & Metabolism, 26, 69–74. 10.1016/j.tem.2014.11.004 25497348

[mbo3810-bib-0091] Oshima, K. (2013). Treg induction by a rationally selected mixture of Clostridia strains from the human microbiota. Gut Microbes, 500, 232–238. 10.1038/nature12331 23842501

[mbo3810-bib-0092] Oursler, M. J. , Osdoby, P. , Pyfferoen, J. , Riggs, B. L. , & Spelsberg, T. C. (1991). Avian osteoclasts as estrogen target cells. Proceedings of the National Academy of Sciences of the United States of America, 88, 6613–6617. 10.1073/pnas.88.15.6613 1907373 PMC52137

[mbo3810-bib-0093] Ozono, K. , Liao, J. , Kerner, S. A. , Scott, R. A. , & Pike, J. W. (1990). The vitamin D‐responsive element in the human osteocalcin gene. Association with a nuclear proto‐oncogene enhancer. Journal of Biological Chemistry, 265, 21881. 2174889

[mbo3810-bib-0094] Parvaneh, K. , Jamaluddin, R. , Karimi, G. , & Erfani, R. (2014). Effect of probiotics supplementation on bone mineral content and bone mass density. The Scientific World Journal, 2014, 595962. 24587733 10.1155/2014/595962 PMC3920759

[mbo3810-bib-0095] Prates, T. P. , Taira, T. M. , Holanda, M. C. , Bignardi, L. A. , Salvador, S. L. , Zamboni, D. S. , … Fukada, S. Y. (2014). NOD2 contributes to porphyromonas gingivalis–induced bone resorption. Journal of Dental Research, 93, 1155–1162. 10.1177/0022034514551770 25239844 PMC4293770

[mbo3810-bib-0096] Qin, J. , Li, Y. , Cai, Z. , Li, S. , Zhu, J. , Zhang, F. , … Shen, D. (2012). A metagenome‐wide association study of gut microbiota in type 2 diabetes. Nature, 490, 55 10.1038/nature11450 23023125

[mbo3810-bib-0097] Queipoortuño, M. I. , Seoane, L. M. , Murri, M. , Pardo, M. , Gomezzumaquero, J. M. , Cardona, F. , … Tinahones, F. J. (2013). Gut microbiota composition in male rat models under different nutritional status and physical activity and its association with serum leptin and ghrelin levels. PLoS ONE, 8, e65465 10.1371/journal.pone.0065465 23724144 PMC3665787

[mbo3810-bib-0098] Redlich, K. , & Smolen, J. S. (2012). Inflammatory bone loss: Pathogenesis and therapeutic intervention. Nature Reviews Drug Discovery, 11, 234–250. 10.1038/nrd3669 22378270

[mbo3810-bib-0099] Reigstad, C. S. , Salmonson, C. E. , Rd, R. J. , Szurszewski, J. H. , Linden, D. R. , Sonnenburg, J. L. , … Kashyap, P. C. (2015). Gut microbes promote colonic serotonin production through an effect of short‐chain fatty acids on enterochromaffin cells. Faseb Journal, 29, 1395–1403. 10.1096/fj.14-259598 25550456 PMC4396604

[mbo3810-bib-0100] Resta‐Lenert, S. , & Barrett, K. E. (2006). Probiotics and commensals reverse TNF‐alpha‐ and IFN‐gamma‐induced dysfunction in human intestinal epithelial cells. Gastroenterology, 130, 731–746. 10.1053/j.gastro.2005.12.015 16530515

[mbo3810-bib-0101] Ridlon, J. M. , Ikegawa, S. , Alves, J. M. , Zhou, B. , Kobayashi, A. , Iida, T. , … De, A. G. (2013). Clostridium scindens: A human gut microbe with a high potential to convert glucocorticoids into androgens. Journal of Lipid Research, 54, 2437–2449. 10.1194/jlr.M038869 23772041 PMC3735941

[mbo3810-bib-0102] Ridlon, J. M. , Kang, D. J. , Hylemon, P. B. , & Bajaj, J. S. (2014). Bile acids and the gut microbiome. Current Opinion in Gastroenterology, 30, 332–338. 10.1097/MOG.0000000000000057 24625896 PMC4215539

[mbo3810-bib-0103] Roshchina, V. V. (2016). New trends and perspectives in the evolution of neurotransmitters in microbial, plant, and animal cells. Advances in Experimental Medicine & Biology, 874, 25. 26589213 10.1007/978-3-319-20215-0_2

[mbo3810-bib-0104] Rubinstein, M. R. , Wang, X. , Liu, W. , Hao, Y. , Cai, G. , & Han, Y. W. (2013). Fusobacterium nucleatum promotes colorectal carcinogenesis by modulating E‐cadherin/β‐catenin signaling via its FadA adhesin. Cell Host & Microbe, 14, 195–206. 10.1016/j.chom.2013.07.012 23954158 PMC3770529

[mbo3810-bib-0105] Ruiz‐Gaspa, S. , Guanabens, N. , Enjuanes, A. , Peris, P. , Martinez‐Ferrer, A. , de Osaba, M. J. , … Combalia, A. (2010). Lithocholic acid downregulates vitamin D effects in human osteoblasts. European Journal of Clinical Investigation, 40, 25–34. 10.1111/j.1365-2362.2009.02230.x 20055894

[mbo3810-bib-0106] Sandoval, D. A. , & D'Alessio, D. A. (2015). Physiology of proglucagon peptides: Role of glucagon and GLP‐1 in health and disease. Physiological Reviews, 95, 513–548. 10.1152/physrev.00013.2014 25834231

[mbo3810-bib-0107] Schnorr, S. L. , Candela, M. , Rampelli, S. , Centanni, M. , Consolandi, C. , Basaglia, G. , … Severgnini, M. (2014). Gut microbiome of the Hadza hunter‐gatherers. Nature Communications, 5, 1–12. 10.1038/ncomms4654 PMC3996546 24736369

[mbo3810-bib-0108] Schwarzer, M. , Makki, K. , Storelli, G. , Machuca‐Gayet, I. , Srutkova, D. , Hermanova, P. , … Heddi, A. (2017). Lactobacillus plantarum strain maintains growth of infant mice during chronic undernutrition. Science, 351, 854–857. 10.1126/science.aad8588 26912894

[mbo3810-bib-0109] Scott, M. M. , Lachey, J. L. , Sternson, S. M. , Lee, C. E. , Elias, C. F. , Friedman, J. M. , & Elmquist, J. K. (2009). Leptin targets in the mouse brain. Journal of Comparative Neurology, 514, 518–532. 10.1002/cne.22025 19350671 PMC2710238

[mbo3810-bib-0110] Shen, Q. , & Christakos, S. (2005). The vitamin D receptor, Runx2, and the Notch signaling pathway cooperate in the transcriptional regulation of osteopontin. Journal of Biological Chemistry, 280, 40589–40598. 10.1074/jbc.M504166200 16195230

[mbo3810-bib-0111] Sims, N. A. , Clément‐Lacroix, P. , Minet, D. , Fraslon‐Vanhulle, C. , Gaillard‐Kelly, M. , Resche‐Rigon, M. , & Baron, R. (2003). A functional androgen receptor is not sufficient to allow estradiol to protect bone after gonadectomy in estradiol receptor‐deficient mice. Journal of Clinical Investigation, 111, 1319–1327. 10.1172/JCI200317246 12727923 PMC154447

[mbo3810-bib-0112] Sjogren, K. , Engdahl, C. , Henning, P. , Lerner, U. H. , Tremaroli, V. , Lagerquist, M. K. , … Ohlsson, C. (2012). The gut microbiota regulates bone mass in mice. Journal of Bone and Mineral Research, 27, 1357–1367. 10.1002/jbmr.1588 22407806 PMC3415623

[mbo3810-bib-0113] Smiricky‐Tjardes, M. R. , Grieshop, C. M. , Flickinger, E. A. , Bauer, L. L. , & Jr, F. G. (2003). Dietary galactooligosaccharides affect ileal and total‐tract nutrient digestibility, ileal and fecal bacterial concentrations, and ileal fermentative characteristics of growing pigs. Journal of Animal Science, 81, 2535–2545. 10.2527/2003.81102535x 14552381

[mbo3810-bib-0114] Smith, P. M. , Howitt, M. R. , Panikov, N. , Michaud, M. , Gallini, C. A. , Bohloolyy, M. , … Garrett, W. S. (2013). The microbial metabolites, short chain fatty acids, regulate colonic Treg cell homeostasis. Science, 341, 569–573. 10.1126/science.1241165 23828891 PMC3807819

[mbo3810-bib-0115] Smith, B. J. , Lerner, M. R. , Bu, S. Y. , Lucas, E. A. , Hanas, J. S. , Lightfoot, S. A. , … Brackett, D. J. (2006). Systemic bone loss and induction of coronary vessel disease in a rat model of chronic inflammation. Bone, 38, 378–386. 10.1016/j.bone.2005.09.008 16256450

[mbo3810-bib-0116] Smith, E. A. , & Macfarlane, G. T. (1997). Dissimilatory amino Acid metabolism in human colonic bacteria. Anaerobe, 3, 327–337. 10.1006/anae.1997.0121 16887608

[mbo3810-bib-0117] Song, L. , Liu, M. , Ono, N. , Richard, F. , Kronenberg, H. M. , & Guo, J. (2012). Loss of wnt/beta‐catenin signaling causes cell fate shift of preosteoblasts from osteoblasts to adipocytes. Journal of Bone and Mineral Research, 27, 2344–2358. 10.1002/jbmr.1694 22729939 PMC3474875

[mbo3810-bib-0118] Souza, J. A. , Medeiros, M. C. , Rocha, F. R. , de Aquino, S. G. , Ávila‐Campos, M. J. , Spolidorio, L. C. , … Rossa, C. (2016). Role of NOD2 and RIP2 in host‐microbe interactions with Gram‐negative bacteria: Insights from the periodontal disease model. Innate Immunity, 22, 598–611. 10.1177/1753425916666652 27605548 PMC6525631

[mbo3810-bib-0119] Stefka, A. T. , Feehley, T. , Tripathi, P. , Qiu, J. , Mccoy, K. , Mazmanian, S. K. , … Theriault, B. R. (2014). Commensal bacteria protect against food allergen sensitization. Proceedings of the National Academy of Sciences, 111, 13145–13150. 10.1073/pnas.1412008111 PMC4246970 25157157

[mbo3810-bib-0120] Storelli, G. , Defaye, A. , Erkosar, B. , Hols, P. , Royet, J. , & Leulier, F. (2011). Lactobacillus plantarum promotes Drosophila systemic growth by modulating hormonal signals through TOR‐dependent nutrient sensing. Cell Metabolism, 14, 403–414. 10.1016/j.cmet.2011.07.012 21907145

[mbo3810-bib-0121] Sugita, S. , Kawazoe, Y. , Imai, A. , Yamada, Y. , Horie, S. , & Mochizuki, M. (2012). Inhibition of Th17 differentiation by anti‐TNF‐alpha therapy in uveitis patients with Behçet's disease. Arthritis Research & Therapy, 14, R99 10.1186/ar3824 22546542 PMC3446476

[mbo3810-bib-0122] Sun, L. , Yu, Z. J. , Ye, X. W. , Zou, S. R. , Li, H. X. , Yu, D. X. , … Clément, K. (2010). A marker of endotoxemia is associated with obesity and related metabolic disorders in apparently healthy Chinese. Diabetes Care, 33, 1925–1932. 10.2337/dc10-0340 20530747 PMC2928335

[mbo3810-bib-0123] Sutton, A. L. M. , & Macdonald, P. N. (2003). Vitamin D: More than a “Bone‐a‐Fide” hormone. Molecular Endocrinology, 17, 777 10.1210/me.2002-0363 12637589

[mbo3810-bib-0124] Tai, P. , Wang, J. , Jin, H. , Song, X. , Yan, J. , Kang, Y. , … Chen, X. (2008). Induction of regulatory T cells by physiological level estrogen. Journal of Cellular Physiology, 214, 456–464. 10.1002/(ISSN)1097-4652 17654501

[mbo3810-bib-0125] Takemura, N. , Okubo, T. , & Sonoyama, K. (2010). Lactobacillus plantarum strain No. 14 reduces adipocyte size in mice fed high‐fat diet. Experimental Biology & Medicine, 235, 849–856. 10.1258/ebm.2010.009377 20558839

[mbo3810-bib-0126] Tashiro, K. , Abe, T. , Oue, N. , Yasui, W. , & Ryoji, M. (2004). Characterization of vitamin D‐mediated induction of the CYP 24 transcription. Molecular & Cellular Endocrinology, 226, 27–32. 10.1016/j.mce.2004.07.012 15489002

[mbo3810-bib-0127] Tashiro, K. , Ishii, C. , & Ryoji, M. (2007). Role of distal upstream sequence in vitamin D‐induced expression of human CYP24 gene. Biochemical and Biophysical Research Communications, 358, 259–265. 10.1016/j.bbrc.2007.04.103 17475215

[mbo3810-bib-0128] Thangaraju, M. , Karunakaran, S. K. , Itagaki, S. , Gopal, E. , Elangovan, S. , Prasad, P. D. , & Ganapathy, V. (2010). Transport via SLC5A8 with subsequent inhibition of histone deacetylases HDAC1 and HDAC3 underlies the antitumor activity of 3‐bromopyruvate. Cancer, 115, 4655–4666. 10.1002/cncr.24532 PMC2782911 19637353

[mbo3810-bib-0129] Tomkinson, A. , Gevers, E. F. , Wit, J. M. , Reeve, J. , & Noble, B. S. (1998). The role of estrogen in the control of rat osteocyte apoptosis. Journal of Bone & Mineral Research, 13, 1243–1250. 10.1359/jbmr.1998.13.8.1243 9718192

[mbo3810-bib-0130] Tsavkelova, E. A. , Siu, K. , Cherdyntseva, T. A. , & Netrusov, A. I. (2006). Hormones and hormone‐like substances of microorganisms: A review. Applied Biochemistry & Microbiology, 42, 229–235. 10.1134/S000368380603001X 16878539

[mbo3810-bib-0131] Tyagi, A. M. , Mansoori, M. N. , Srivastava, K. , Khan, M. P. , Kureel, J. , Dixit, M. , … Singh, D. (2014). Enhanced immunoprotective effects by anti‐IL‐17 antibody translates to improved skeletal parameters under estrogen deficiency compared with anti‐RANKL and anti‐TNF‐α antibodies. Journal of Bone & Mineral Research, 29, 1981–1992. 10.1002/jbmr.2228 24677326

[mbo3810-bib-0132] Vrieze, A. , Out, C. , Fuentes, S. , Jonker, L. , Reuling, I. , Kootte, R. S. , … Romijn, J. A. (2014). Impact of oral vancomycin on gut microbiota, bile acid metabolism, and insulin sensitivity. Journal of Hepatology, 60, 824–831. 10.1016/j.jhep.2013.11.034 24316517

[mbo3810-bib-0133] Wade, P. R. , Chen, J. , Jaffe, B. , Kassem, I. S. , Blakely, R. D. , & Gershon, M. D. (1996). Localization and function of a 5‐HT transporter in crypt epithelia of the gastrointestinal tract. Journal of Neuroscience, 16, 2352–2364. 10.1523/JNEUROSCI.16-07-02352.1996 8601815 PMC3327288

[mbo3810-bib-0134] Wallace, T. C. , Marzorati, M. , Spence, L. , Weaver, C. M. , & Williamson, P. S. (2017). New frontiers in fibers: Innovative and emerging research on the gut microbiome and bone health. Journal of the American College of Nutrition, 36, 218–222. 10.1080/07315724.2016.1257961 28318400

[mbo3810-bib-0135] Wang, Y. , Cheng, Z. , Elalieh, H. Z. , Nakamura, E. , Nguyen, M. T. , Mackem, S. , … Chang, W. (2011). IGF‐1R signaling in chondrocytes modulates growth plate development by interacting with the PTHrP/Ihh pathway. Journal of Bone & Mineral Research, 26, 1437–1446. 10.1002/jbmr.359 21312270 PMC3530140

[mbo3810-bib-0136] Wang, X. , Yang, Y. , & Huycke, M. M. (2017). Commensal‐infected macrophages induce dedifferentiation and reprogramming of epithelial cells during colorectal carcinogenesis. Oncotarget, 8, 102176–102190. 29254234 10.18632/oncotarget.22250 PMC5731944

[mbo3810-bib-0137] Weaver, C. M. (2015). Diet, gut microbiome, and bone health. Current Osteoporosis Reports, 13, 125–130. 10.1007/s11914-015-0257-0 25616772 PMC4996260

[mbo3810-bib-0138] Weaver, C. M. , Gordon, C. M. , Janz, K. F. , Kalkwarf, H. J. , Lappe, J. M. , Lewis, R. , … Zemel, B. S. (2016). The National Osteoporosis Foundation's position statement on peak bone mass development and lifestyle factors: A systematic review and implementation recommendations. Osteoporosis International, 27, 1281–1386. 10.1007/s00198-015-3440-3 26856587 PMC4791473

[mbo3810-bib-0139] Weitzmann, M. N. , Roggia, C. , Toraldo, G. , Weitzmann, L. , & Pacifici, R. (2002). Increased production of IL‐7 uncouples bone formation from bone resorption during estrogen deficiency. Journal of Clinical Investigation, 110, 1643–1650. 10.1172/JCI0215687 12464669 PMC151629

[mbo3810-bib-0140] Wells, J. E. , & Hylemon, P. B. (2000). Identification and characterization of a bile acid 7alpha‐dehydroxylation operon in Clostridium sp. strain TO‐931, a highly active 7alpha‐dehydroxylating strain isolated from human feces. Applied and Environment Microbiology, 66, 1107–1113. 10.1128/AEM.66.3.1107-1113.2000 PMC91949 10698778

[mbo3810-bib-0141] Wichmann, A. , Allahyar, A. , Greiner, T. U. , Plovier, H. , Lundén, G. Ö. , Larsson, T. , … Bäckhed, F. (2013). Microbial modulation of energy availability in the colon regulates intestinal transit. Cell Host & Microbe, 14, 582–590. 10.1016/j.chom.2013.09.012 24237703

[mbo3810-bib-0142] Wu, S. , Morin, P. J. , Maouyo, D. , & Sears, C. L. (2003). Bacteroides fragilis enterotoxin induces c‐Myc expression and cellular proliferation. Gastroenterology, 124, 392–400. 10.1053/gast.2003.50047 12557145

[mbo3810-bib-0143] Yadav, V. K. , Oury, F. , Suda, N. , Liu, Z. W. , Gao, X. B. , Confavreux, C. , … Guo, X. E. (2009). A serotonin‐dependent mechanism explains the leptin regulation of bone mass, appetite, and energy expenditure. Cell, 138, 976–989. 10.1016/j.cell.2009.06.051 19737523 PMC2768582

[mbo3810-bib-0144] Yadav, V. K. , Ryu, J. H. , Suda, N. , Tanaka, K. F. , Gingrich, J. A. , Schütz, G. , … Karsenty, G. (2008). Lrp5 controls bone formation by inhibiting serotonin synthesis in the duodenum. Cell, 135, 825–837. 10.1016/j.cell.2008.09.059 19041748 PMC2614332

[mbo3810-bib-0145] Yakar, S. , Rosen, C. J. , Beamer, W. G. , Ackert‐ Bicknell, C. L. , Wu, Y. , Liu, J. L. , … LeRoith, D. (2002). Circulating levels of IGF‐I directly regulate bone growth and density. Journal of Clinical Investigation, 110, 771–781. 10.1172/JCI0215463 12235108 PMC151128

[mbo3810-bib-0146] Yan, J. , Herzog, J. W. , Tsang, K. , Brennan, C. A. , Bower, M. A. , Garrett, W. S. , … Charles, J. F. (2016). Gut microbiota induce IGF‐1 and promote bone formation and growth. Proceedings of the National Academy of Sciences of the United States of America, 113, E7554–E7563. 10.1073/pnas.1607235113 27821775 PMC5127374

[mbo3810-bib-0147] Yang, S. , Takahashi, N. T. , Sato, N. , Takahashi, M. , Mogi, M. , Uematsu, T. , … Akira, S. (2005). Muramyl dipeptide enhances osteoclast formation induced by lipopolysaccharide, IL‐1{alpha}, and TNF‐{alpha} through nucleotide‐binding oligomerization domain 2‐mediated signaling in osteoblasts. Journal of Immunology, 175, 1956–1964. 10.4049/jimmunol.175.3.1956 16034140

[mbo3810-bib-0148] Yang, Y. , Wang, X. , Huycke, T. , Moore, D. R. , Lightfoot, S. A. , & Huycke, M. M. (2013). Colon macrophages polarized by commensal bacteria cause colitis and cancer through the bystander effect. Translational Oncology, 6, 596–606. 10.1593/tlo.13412 24151540 PMC3799201

[mbo3810-bib-0149] Yang, Y. , Wang, X. , Moore, D. R. , Lightfoot, S. A. , & Huycke, M. M. (2012). TNF‐α mediates macrophage‐induced bystander effects through Netrin‐1. Cancer Research, 72, 5219–5229. 10.1158/0008-5472.CAN-12-1463 22915753 PMC3473172

[mbo3810-bib-0150] Yano, J. M. , Yu, K. , Donaldson, G. P. , Shastri, G. G. , Ann, P. , Ma, L. , … Hsiao, E. Y. (2015). Indigenous bacteria from the gut microbiota regulate host serotonin biosynthesis. Cell, 161, 264–276. 10.1016/j.cell.2015.02.047 25860609 PMC4393509

[mbo3810-bib-0151] Yuan, F. L. , Li, X. , Lu, W. G. , Xu, R. S. , Zhao, Y. Q. , Li, C. W. , … Chen, F. H. (2010). Regulatory T cells as a potent target for controlling bone loss. Biochemical & Biophysical Research Communications, 402, 173–176. 10.1016/j.bbrc.2010.09.120 20920469

[mbo3810-bib-0152] Yun, K. L. , & Gordon, J. I. (2011). Proinflammatory T‐cell responses to gut microbiota promote experimental autoimmune encephalomyelitis. Proceedings of the National Academy of Sciences of the United States of America, 108, 4615–4622. 20660719 10.1073/pnas.1000082107 PMC3063590

[mbo3810-bib-0153] Zaiss, M. M. , Axmann, R. , Zwerina, J. , Polzer, K. , Gückel, E. , Skapenko, A. , … Schett, G. (2007). Treg cells suppress osteoclast formation: A new link between the immune system and bone. Arthritis & Rheumatology, 56, 4104–4112. 10.1002/art.23138 18050211

[mbo3810-bib-0154] Zaiss, M. M. , Sarter, K. , Hess, A. , Engelke, K. , Böhm, C. , Nimmerjahn, F. , … David, J. P. (2010). Increased bone density and resistance to ovariectomy‐induced bone loss in FoxP3‐transgenic mice based on impaired osteoclast differentiation. Arthritis & Rheumatism, 62, 2328–2338. 10.1002/art.27535 20506516

[mbo3810-bib-0155] Zhang, J. , Fu, Q. , Ren, Z. , Wang, Y. , Wang, C. , Shen, T. , … Wu, L. (2014). Changes of serum cytokines‐related Th1/Th2/Th17 concentration in patients with postmenopausal osteoporosis. Gynecological Endocrinology, 31, 183–190. 25384921 10.3109/09513590.2014.975683

[mbo3810-bib-0156] Zhang, J. , Motyl, K. J. , Irwin, R. , Macdougald, O. A. , Britton, R. A. , & Mccabe, L. R. (2015). Loss of bone and Wnt10b expression in male type 1 diabetic mice is blocked by the probiotic lactobacillus reuteri. Endocrinology, 156, 3169–3182. 10.1210/EN.2015-1308 26135835 PMC4541610

